# Deciphering the Impact of Nucleosides and Nucleotides on Copper Ion and Dopamine Coordination Dynamics

**DOI:** 10.3390/ijms25179137

**Published:** 2024-08-23

**Authors:** Patrycja Sadowska, Wojciech Jankowski, Romualda Bregier-Jarzębowska, Piotr Pietrzyk, Renata Jastrząb

**Affiliations:** 1 Faculty of Chemistry, Adam Mickiewicz University in Poznań, Uniwersytetu Poznańskiego 8, 61-614 Poznań, Poland; patrycja.sadowska@amu.edu.pl (P.S.); wojciech.jankowski@amu.edu.pl (W.J.); bregier@amu.edu.pl (R.B.-J.); 2 Faculty of Chemistry, Jagiellonian University, Gronostajowa 2, 30-387 Kraków, Poland; piotr.pietrzyk@uj.edu.pl

**Keywords:** coordination chemistry, copper(II) ion complexes, dopamine, purine nucleotides, potentiometric studies, spectroscopic studies, molecular modeling and DFT studies

## Abstract

The mode of coordination of copper(II) ions with dopamine (DA, L) in the binary, as well as ternary systems with Ado, AMP, ADP, and ATP (L′) as second ligands, was studied with the use of experimental—potentiometric and spectroscopic (VIS, EPR, NMR, IR)—methods and computational—molecular modeling and DFT—studies. In the Cu(II)/DA system, depending on the pH value, the active centers of the ligand involved in the coordination with copper(II) ions changed from nitrogen and oxygen atoms (CuH(DA)^3+^, Cu(DA)^2+^), via nitrogen atoms (CuH_2_(DA)_2_^4+^), to oxygen atoms at strongly alkaline pH (Cu(DA)_2_^2+^). The introduction of L′ into this system changed the mode of interaction of dopamine from oxygen atoms to the nitrogen atom in the hydroxocomplexes formed at high pH values. In the ternary systems, the ML′-L (non-covalent interaction) and ML′HxL, ML′L, and ML′L(OH)x species were found. In the Cu(II)/DA/AMP or ATP systems, mixed forms were formed up to a pH of around 9.0; above this pH, only Cu(II)/DA complexes occurred. In contrast to systems with AMP and ATP, ternary species with Ado and ADP occurred in the whole pH range at a high concentration, and moreover, binary complexes of Cu(II) ions with dopamine did not form in the detectable concentration.

## 1. Introduction

Research on biologically active organic compounds, such as catecholamines or small fragments of nucleic acid, is of ongoing interest due to its significant impact on the processes of living organisms and, consequently, on their proper functioning [1,2]. Inspired by nature, scientists undertake a systematic study on the understanding of the mechanisms of their interactions, especially with metal ions, which affect the structure and activity of biomolecules [3,4,5].

**Figure 1:**
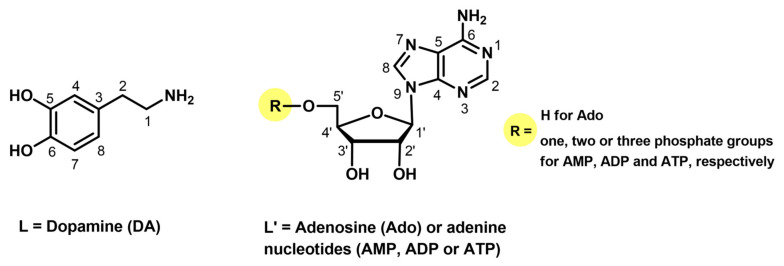
Chemical formulae of the bioligands studied.

As was described earlier [2,6], dopamine (Figure 1) is a bioenergetic catecholamine that acts as a neurotransmitter in the central nervous system. Its abnormal level in the body is responsible for the occurrence of pathological conditions such as Parkinson’s disease and schizophrenia [2,7,8]. Dopamine (DA) is also easily oxidized and subsequently self-polymerizes in contact with air, oxidants (for example Cu^2+^ ions), light, and a high-pH environment [9,10,11]. 

Nucleosides and nucleotides are multifunctional bioligands. As a fragments of nucleic acids, these compounds are extremely important for living organisms [12,13,14]. They are involved in genetic information and intramolecular energy transfer, being components of coenzymes [5,12]. Adenosine (Ado) is a major signaling nucleoside that is an essential component of energy production used in all living cells. Adenosine also plays key roles in regulation of various physiological functions (e.g., immune responses, neuronal activity, and energy balance) [15,16]. On the other hand, adenosine 5′-monophosphate (AMP) is responsible for energy homeostasis and activating the enzyme protein kinase, which stimulates glucose uptake and the β-oxidation of fatty acids, as well as inhibiting cholesterol synthesis [17,18]. Adenosine 5′-diphosphate (ADP) plays an important role in vascular and cellular responses and is immediately released after tissue injury, mainly from platelets [19,20]. In turn, adenosine 5′-triphosphate (ATP), being an extracellular signaling molecule, acts as a neurotransmitter in the peripheral and central nervous systems [3,21]. Each living cell also contains metal ions, including Cu(II) ions, which can be the active centers of several enzymes involved in processes occurring at the cellular level, for example, hemoglobin formation, drug metabolism, and catecholamine biosynthesis [22]. The copper(II) ion is considered a borderline Lewis acid and favors ligands with hard -N and -O donor atoms [23,24] (such as catecholamines and DNA fragments). It is currently known that reactions of metal ions (mainly transition ones) with biomolecules determine the character of processes taking place in living organisms [12,25].

Given the biological importance of the above compounds, the study of the reaction of their complexation with Cu(II) ions is part of the current trends in chemical research [7,10,26,27,28], e.g., in the coordination chemistry of neurotransmitters, and in determining the role of transition metal ions in the signal dysfunction [29]. There are papers in the literature that describe the ability of dopamine (DA) and nucleotides to form binary as well as ternary complexes with metal ions [28,30,31,32,33,34,35,36]. However, we are keeping with this theme, in order to have new experimental data on the interactions in the conditions simulating biological systems, as well as to update previous literature information for better understanding the processes taking place in living organisms.

In the paper presented, we have attempted to answer the question of what is the mode of coordination in the complexes formed in the model Cu(II)/dopamine and Cu(II)/dopamine/nucleoside (or nucleotide) systems.

The chemical formulae of ligands studied are presented in Figure 1. 

## 2. Results and Discussion

Potential coordination centers of the ligands studied (Figure 1) are two phenolic hydroxyl groups and a terminal amino group from dopamine, as well as the endocyclic nitrogen atoms N(1) or N(7) from the adenine ring in adenosine, AMP, ADP, and ATP as well as the phosphate groups from nucleotides [36,37,38]. The protonation constants of dopamine as the overall stability constants (log*β)* of the complexes formed in the binary Cu(II)/DA system had been determined earlier [6,32,37,39]. We verified these data under our experimental conditions, obtaining results in good agreement with the literature (Table 1). Furthermore, this part of the work could not be omitted, as the data obtained were then used to analyze mixed complexes and determine the impact of nucleosides and nucleotides on the coordination of copper ion and dopamine.

As was reported previously, there is a significant problem with the determination of the value of the protonation constant of H(DA)^+^, mainly in aqueous solution with an ionic strength under 0.1 M at the temperature of 20 °C [37]. This is the reason why in some papers, the authors do not specify this value and the data in others are often unstable [6,32,37,39,40,41]. Importantly, this fact made it impossible to calculate the values log*K_e_* for some protonated binary and ternary complexes forming in the analyzed systems. Values of protonation constants discussing Ado and nucleotides as well as overall stability constants (log*β*) of the complexes formed in the binary Cu(II)/Ado, Cu(II)/AMP, Cu(II)/ADP, and Cu(II)/ATP systems have been presented already by our group [36,42,43,44] and were used to determine the overall stability constants of mixed-ligand complexes. Moreover, as was described earlier, there is a problem with the unambiguous determination of the order of dissociation of dopamine. The first hypothesis assumes deprotonation of para-phenolate, followed by alkylammonium cation and finally the meta-phenolate [37,45]. In other papers, this dissociation scheme is questioned, and the authors state that the ammonium cation dissociates at the beginning, before para-phenolate, because it is more acidic than it [46,47,48]. However, the first scheme of deprotonation dopamine is more commonly suggested in the literature [6,31,37,45,49,50], so we have also adopted it in our considerations.

### 2.1. Interactions in the Cu(II)/DA Binary System

Using the HYPERQUAD program, the formation of the following forms was found in the Cu(II)/DA binary system: CuH(DA)^3+^, Cu(DA)^2+^, CuH_2_(DA)_2_^4+^, CuH(DA)_2_^3+^, and Cu(DA)_2_^2+^. The determined values of overall stability constants (log*β*) as well as equilibrium constants (log*K_e_*) of these complexes are presented in Table 1, being in good agreement with the literature [31,32,39].

**Figure 2:**
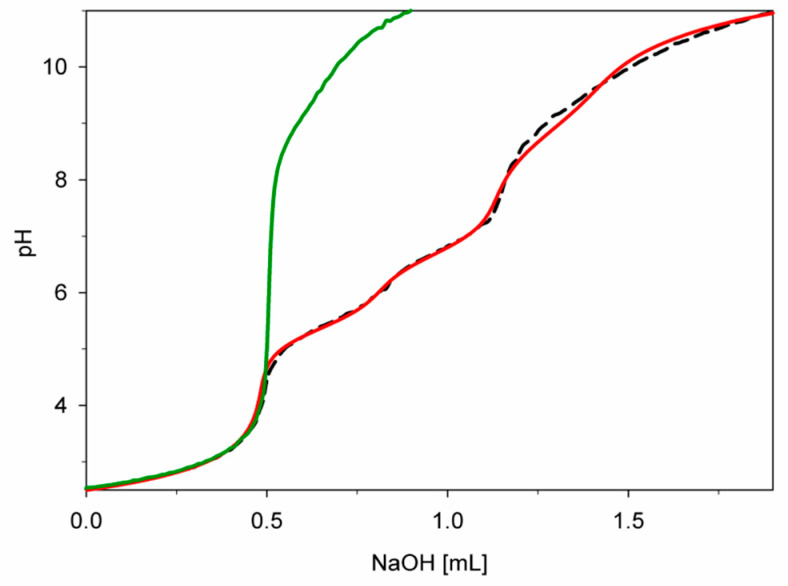
Experimental and simulated titration curves for the Cu(II)/DA system; green—experimental curve (complexes formation was not taken into account); red—experimental curve, black—simulated curve (complexes formation was taken into account); C_Cu_^2+^ = 1 × 10^−3^ M, C_DA_ = 4 × 10^−3^ M.

The correct choice of model was also confirmed by the slight standard deviation of stability constants (log*β*) and good overlapping of the experimental titration curve with that obtained from the computer simulation, where all creating forms were considered. Moreover, a completely different course of this curve from pH above 4.5 compared to the course of the free dopamine curve indicates the formation of complex forms from this pH value (Figure 2).

**Figure 3:**
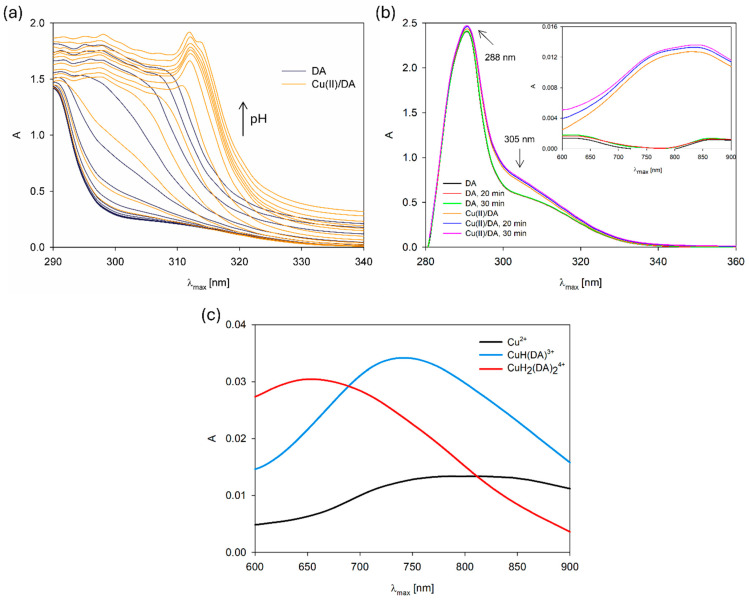
UV–VIS spectra of the (**a**) DA and Cu(II)/DA system depending on the pH, C_Cu_^2+^ = 1 × 10^−5^ M, C_DA_ = 4 × 10^−5^ M; (**b**) DA and Cu(II)/DA system at pH 4.8 depending on the time in the range of 280 nm–900 nm; (**c**) Cu(II)/DA system. C_Cu_^2+^ = 1 × 10^−3^ M, C_DA_ = 4 × 10^−3^ M.

For additional confirmation of the complexation reaction, we compared the UV–VIS spectra of the free DA and Cu(II)/DA system in terms of pH value (Figure 3a). The band shift at 310 nm and variation in its position with the increase in pH, in comparison with the free dopamine band, also points to the formation of Cu(II) complexes [6]. Moreover, we made the UV–VIS spectra of the formation of the CuH(DA)^3+^ species in the reference system as a function change over time (Figure 3b). As was observed, the intensity and position single absorption band at about 288 nm of free dopamine did not change in time (30 min) [26], but after the addition of metal ions, the new band at about 305 nm appeared, which suggests changes in the electronic structure of the dopamine and interaction with Cu^2+^. Furthermore, the presence of a new complex form was also confirmed by the differences in the absorption intensity in the range from 600 nm to 900 nm at the ligand and complex spectra (Figure 3c).

**Figure 4:**
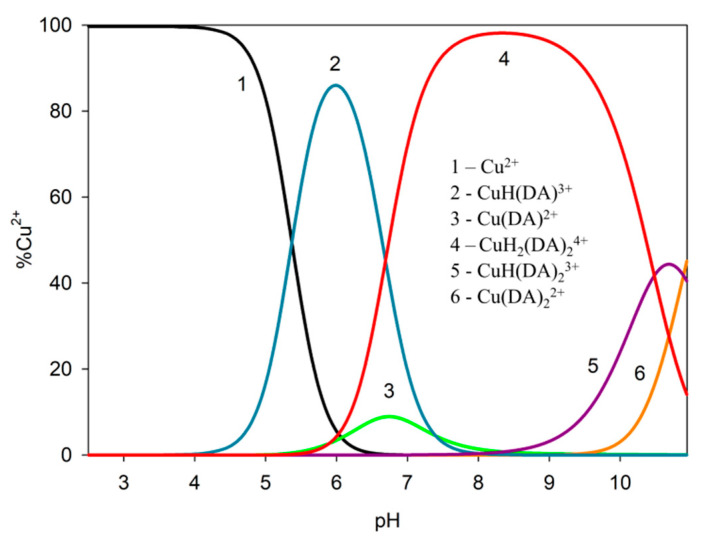
Distribution diagram for the Cu(II)/DA system; percentage of the species refers to total Cu(II); C_Cu_^2+^ = 1 × 10^−3^ M, C_DA_ = 4 × 10^−3^ M.

**Figure 5:**
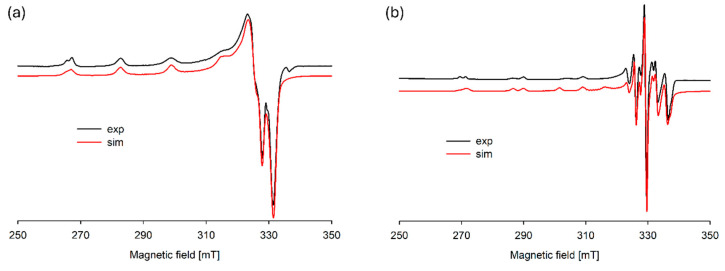
Experimental and simulated EPR spectra for the Cu(II)/DA system at (**a**) pH 6.0 and (**b**) pH 8.0; C_Cu_^2+^ = 1 × 10^−3^ M, C_DA_ = 4 × 10^−3^ M.

In the studied system, the reaction of complexation started at a pH above 4.0 with the formation of the protonated CuH(DA)^3+^ complex (logβ = 23.93), for which the maximum concentration was observed at a pH of about 6.0, where 85% of copper(II) ions participated in coordination (Figure 4). The position of the d-d transition band in the VIS spectrum (λ_max_~740 nm, Figure 3c) as well as EPR parameter values (g_||_ = 2.318, A_||_ = 171 × 10^−4^ cm^−1^, Figure 5a, Table 2) assigned to this species indicated the presence of {1N,1O} chromophore [51,52] and participation in coordination with Cu(II) ions of the oxygen atom from the first deprotonated phenolic group and the nitrogen atom from the -NH_2_ group of dopamine.

**Figure 6:**
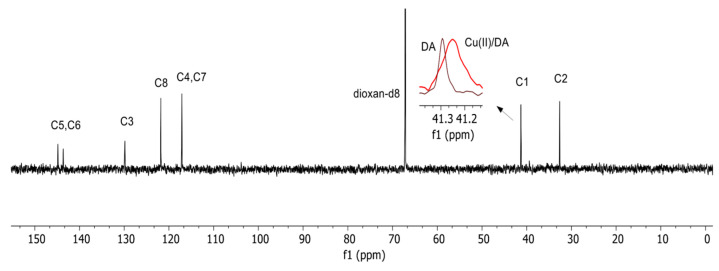
^13^C NMR spectra of the DA and Cu(II)/DA system at pH 6.0; C_Cu_^2+^ = 1 × 10^−3^ M, C_DA_ = 1 × 10^−1^ M.

The involvement of the nitrogen atom from dopamine in the coordination of the CuH(DA)^3+^ complex was also confirmed by the ^13^C NMR spectroscopy (Figure 6). At pH 6.0, where this form dominated, a shift in signal position from the carbon atom (C1) in the vicinity of the amino group (relative to free ligand) was at 0.050 ppm. 

**Figure 7:**
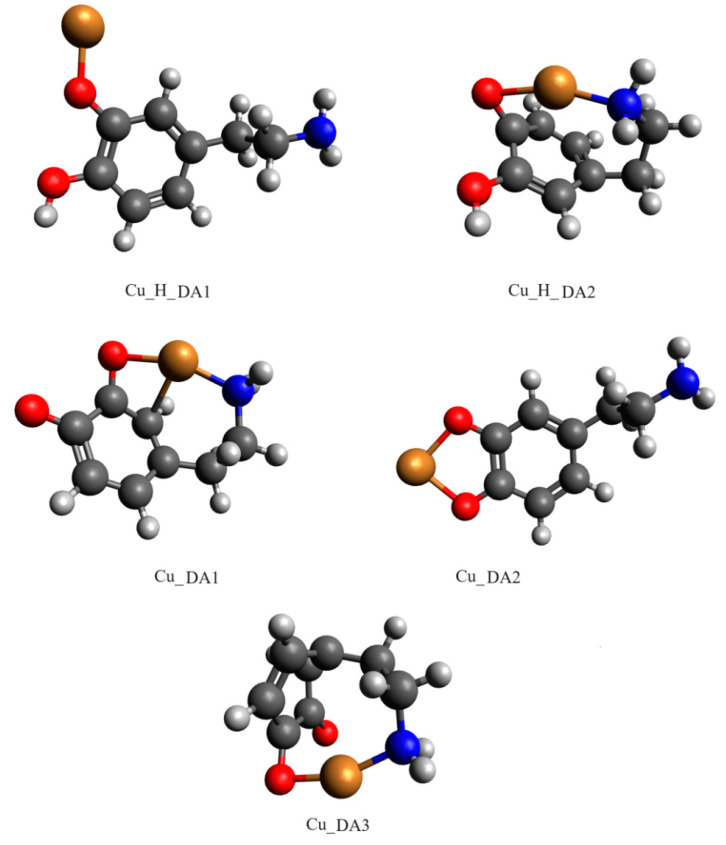
Optimized structures of complexes of dopamine and protonated dopamine with the copper(II) ion; dark grey—carbon atom, light grey—hydrogen atom, red—oxygen atom, blue—nitrogen atom and brown—copper(II) ion.

Computational studies confirmed our proposed mode of coordination. By the obtained results, in the protonated form of the dopamine Cu(II) complex, it coordinated copper with para phenolate and a nitrogen atom (Cu_H_DA2 complex, Figure 7, Chapter: Quantum-chemical calculations in binary Cu(II)/DA system). 

Another complex, i.e., Cu(DA)^2+^ (log*β*=16.53) occurred from pH 6.0 to pH 8.0, binding only about 10% of metal ions (Figure 4). Unfortunately, this complex was also present in the pH range where CuH(DA)^3+^ and CuH_2_(DA)_2_^4+^ complexes are dominant, and therefore it was not possible to determine the mode of coordination in this form using spectroscopic methods; hence, the computational studies were conducted. The obtained results show that in this complex, free dopamine coordinated copper with oxygen from para phenolate and a nitrogen atom, just like in the CuH(DA)^3+^ species (Cu_DA3 complex, Figure 7, Chapter: Quantum-chemical calculations in binary Cu(II)/DA system).

**Figure 8:**
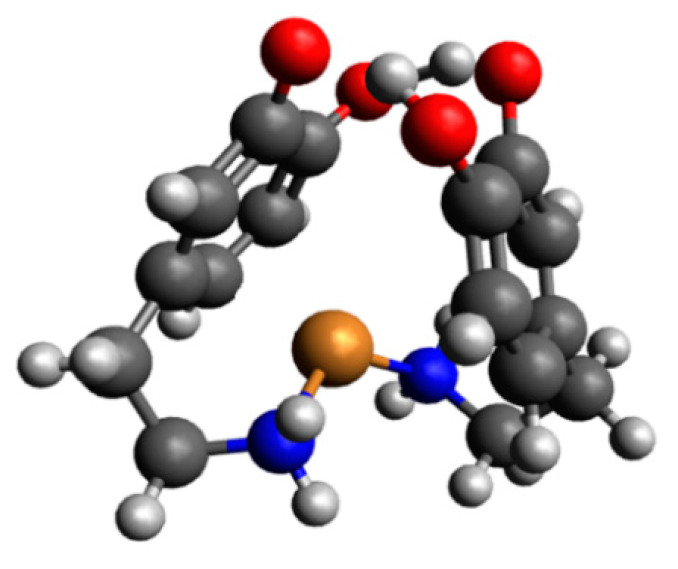
Structure of a complex with the strongest interaction between the two molecules of protonated dopamine and the copper(II) ion (Cu_H_DA2_2_A1); dark grey—carbon atoms, light grey—hydrogen atoms, red—oxygen atoms, blue—nitrogen atoms and brown—copper(II) ion.

In the pH range 6.5–10.5, the dominant species was CuH_2_(DA)_2_^4+^ (log*β* = 45.37) binding close to 100% of copper(II) ions (Figure 4). This form formed an attachment to the anchoring CuH(DA)^3+^ second H(DA) molecule. Unexpectedly, for the species, the absorption maximum λ_max_ = 658 nm (Figure 3c, Table 2) points to the metal bonding only by the two amino groups from two dopamine molecules, chromophore {2N} [53,54]. Moreover, the analysis of EPR spectra at pH 8.0 (Figure 5b, Table 2), indicates the presence of two centers, which may indicate that in the second dopamine molecule in the CuH_2_(DA)_2_ species, although it coordinates through the same nitrogen atom, it has a different symmetry. Additionally, according to computational studies, this type of interaction is energetically more favorable (Cu_H_DA2_2_A1, Figure 8, Chapter: Quantum-chemical calculations in binary Cu(II)/DA system).

**Figure 9:**
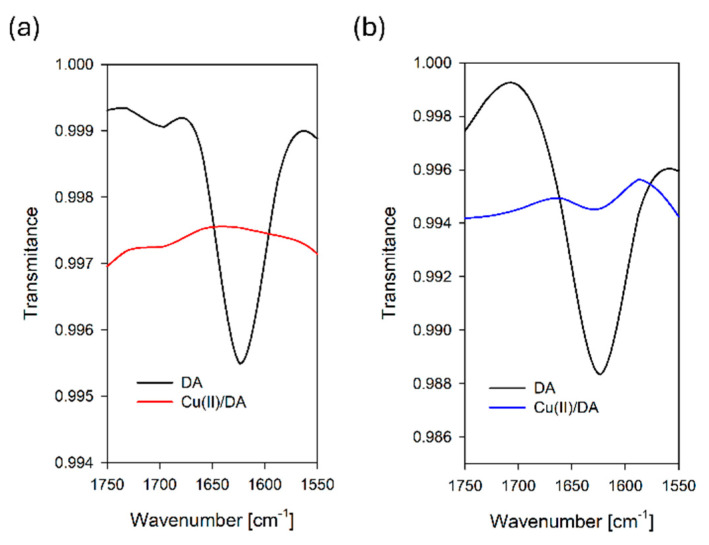
Fragment of FT-IR spectra of the DA and Cu(II)/DA system at (**a**) pH 6.0 and (**b**) pH 8.0; C_Cu_^2+^ = 1 × 10^−3^ M, C_DA_ = 4 × 10^−3^ M.

To further confirm the contribution of nitrogen in the coordination up to pH 8.0, we carried out IR measurements, which in the absence of X-ray crystallography can be helpful for elucidating the way of binding the organic ligands to the metal ions [7]. The IR spectra of the free dopamine and the CuH(DA)^3+^ and CuH_2_(DA)_2_^4+^ complexes exhibited broad bands at about 3400 cm^−1^ (Figure 9) due to phenolic group stretching vibration (ν(OH)), as well as amino group stretching vibration (ν(NH)). The presence of hydrated water molecules renders it difficult to confirm the participation of this band in chelate formation [7]. Conversely, the disappearance of the band due to δ(NH) at 1622 and 1627 cm^−1^ at pH 6.0 and 8.0 (Figure 9), respectively, may indicate the involvement of coordination in these species of the nitrogen atom from dopamine [7,55].

**Figure 10:**
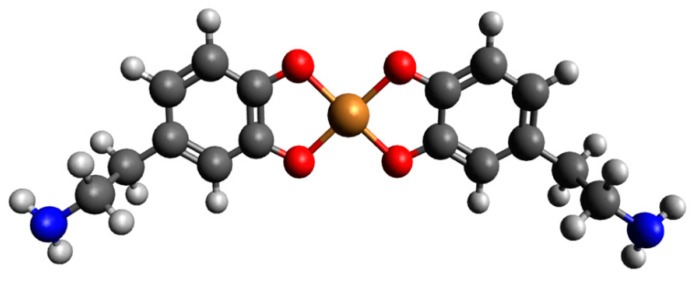
Structure of the complex with the strongest interaction between the two molecules of dopamine and the copper(II) ion (Cu_DA2_3); dark grey—carbon atoms, light grey—hydrogen atoms, red—oxygen atoms, blue—nitrogen atoms and brown—copper(II) ion.

Above pH 9.0, CuH(DA)_2_^3+^ and Cu(DA)_2_^2+^ complexes with log*β* values of 34.89 and 24.00, respectively, were present in the studied system. As with the ML form, also for these species, it was not possible to take spectral studies because in this pH range occurs a mixture of complexes. In addition, above pH 8.0, the dark precipitate was observed in the samples for the ^13^C NMR analysis. Therefore, the only method to determine coordination for these complexes was theoretical calculations. According to computational studies, in the Cu(DA)_2_ complex, the most energetically favorable interactions are for the {4O} chromophore, i.e., copper ions are coordinated by four oxygen atoms from two completely deprotonated dopamine molecules (Cu_DA2_3, Figure 10, Chapter: Quantum-chemical calculations in binary Cu(II)/DA system). As previously established [39], we have confirmed, that in the high pH range, oxygen atoms from the -OH groups of dopamine are preferred in coordination with copper(II). 

#### Quantum-Chemical Calculations in the Binary Cu(II)/DA System

**Figure 11:**
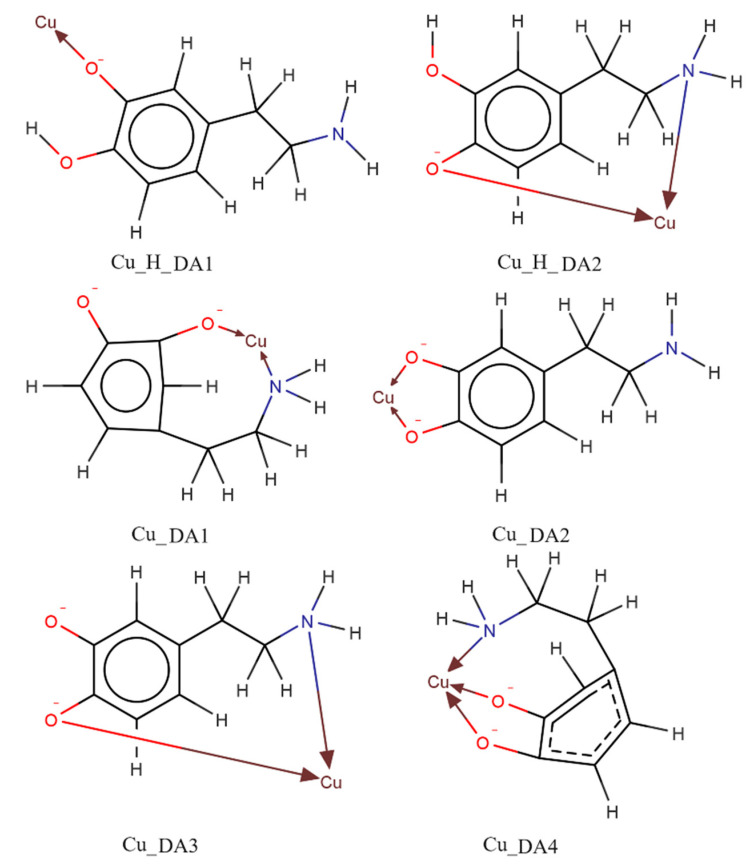
Schemes of a possible interaction between two protonated molecules of dopamine with a copper(II) ion; red—oxygen atoms, blue—nitrogen atoms and brown—copper(II) ions.

Preliminary optimizations for complexes consisting of dopamine or protonated dopamine with copper(II) ion showed that it is not possible to obtain a complex from the interaction scheme Cu_DA4. During calculations, this type of complex converts into the Cu_DA3 scheme of interaction (Figure 11). Calculated energies, sum of monomers, and interaction energy in water are gathered in Table 3.

The obtained results show that the strongest energy interaction between dopamine and copper(II) ion had the complex scheme Cu_DA3. In this complex, dopamine coordinates copper with an oxygen atom from only one of two phenolic groups (oxygen atom from the second phenolic group in not involved in the coordination of the Cu ion) and the nitrogen atom. The interaction energy of such binding was −276.2 kcal/mol. In the protonated form of the dopamine Cu(II) complex, the strongest interaction energy (−208.7 kcal/mol) had the Cu_H_DA2 complex. In this structure, dopamine coordinates copper with oxygen and nitrogen atoms. The optimized structures of all the obtained complexes are depicted in Figure 7. Coordinates of those structures are included in Tables S1 and S2.

The performed calculations for complexes consisting of two protonated molecules of dopamine and copper(II) ion showed that for interaction schemes Cu_H_DA2_1A, Cu_H_DA2_1B, Cu_H_DA2_2A, and Cu_H_DA2_2B, it is not possible to obtain a complex in which dopamine coordinates the copper(II) ion with a nitrogen atom and oxygen atom. The complexes obtained from these interaction schemes had both molecules of dopamine coordinating via the nitrogen atom. Calculated energies, sum of monomers, and interaction energy in water obtained for these complexes are gathered in Table 4.

As shown in Table 4, the strongest interaction energy had complex Cu_H_DA2_2_A1. The calculations allowed us to obtain two complexes from the interaction scheme Cu_H_DA2_2_A, which differed in terms of the orientation of protonated dopamine molecules towards each other. In a complex with the strongest interaction between protonated molecules of dopamine and the copper(II) ion, protonated dopamine molecules had oxygen atoms (with and without hydrogen atoms) close to each other so we observed two hydrogen bonds that additionally stabilized the complex. The lengths of these hydrogen bonds were close to 1.7 Å. The interaction energy in this complex was −356.1 kcal/mol. The obtained structure is depicted in Figure 8. Structures of the rest of the obtained complexes are included in Figure S1. Coordinates of all the obtained structures are included in Tables S3–S6.

Similarly to the case of complexes with two protonated molecules of dopamine with the copper(II) ion for the complex consisting of the two molecules of dopamine and the copper(II) ion for interaction schemes Cu_DA2_1A, Cu_DA2_1B, Cu_DA2_2A, and Cu_DA2_2B, it was not possible to obtain a complex in which dopamine coordinated copper(II) ion via nitrogen and oxygen atoms. Calculated energies, a sum of monomers, and the interaction energy in water obtained for complexes consisting of two molecules of dopamine and copper(II) ion are gathered in Table 5.

In all of the investigated complexes, the strongest interaction energy between the two molecules of dopamine and the copper(II) ion had a complex from the interaction scheme Cu_DA2_3. In the complex from this interaction scheme, both dopamine molecules coordinated copper(II) ions via their oxygen atoms. The energy of such an interaction was −319.1 kcal/mol. The optimized structure of this complex is depicted in Figure 10.

Structures of the rest of the obtained complexes are depicted in Figure S2, and coordinates of all the obtained structures are gathered in Tables S7 and S8. 

### 2.2. Interactions in the Cu(II)/DA/Nucleoside (or Nucleotide) Ternary System 

Overall stability constants of the complexes formed in the ternary Cu(II)/DA/Ado or AMP, ADP, and ATP systems were determined by taking into account the values of protonation constants of ligands and overall stability constants (log*β*) of the complexes formed in the binary Cu(II)/DA, Cu(II)/Ado, Cu(II)/AMP, Cu(II)/ADP, and Cu(II)/ATP (Table 1 and Table 6) systems [36,42,43,44]. From our best knowledge, in the literature, there are no reports on complexation reactions in aqueous solutions in the Cu/DA/Ado or AMP and ADP systems. In 1984, Kiss et al. [56] investigated the Cu(II)/DA/ATP system, where only the Cu(ATP)H(DA) complex (log*β* = 27.59) [56] was found. In our study, besides the Cu(ATP)H(DA) species (log*β* = 27.56), we detected the formation of two, not previously reported, complex forms Cu(ATP)H_3_(DA) and Cu(ATP)H_2_(DA).

**Figure 12:**
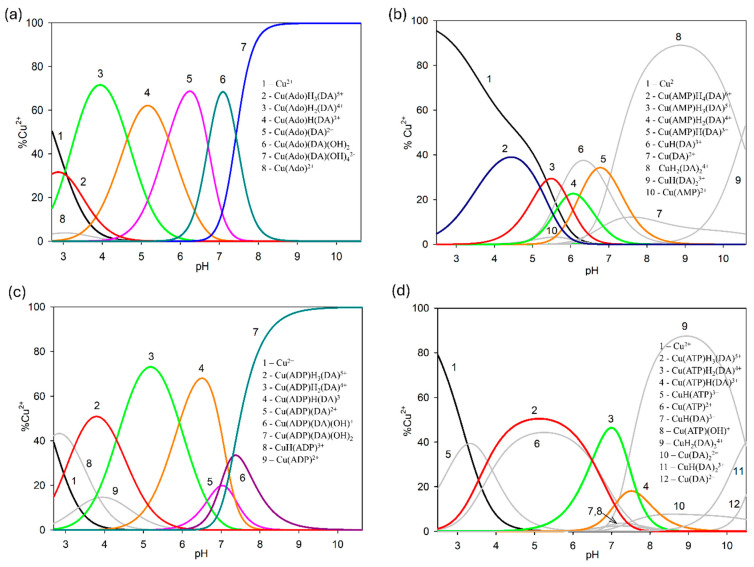
Distribution diagrams for the (**a**) Cu(II)/DA/Ado, (**b**) Cu(II)/DA/AMP, (**c**) Cu(II)/DA/ADP, and (**d**) Cu(II)/DA/ATP systems; percentage of the species refers to total Cu(II); C_Cu_^2+^ = 1 × 10^−3^ M, C_L=L′_ = 2 × 10^−3^ M.

In all Cu(II)/DA/adenosine or adenosine phosphates systems, two types of ternary complexes were found, molecular complexes ML′H_3_L (H_3_DA interacts by non-covalent interactions with anchoring CuL′) as well as protonated ML′H_x_L species. Solely in the Cu(II)/DA/AMP system, the formation of the molecular complex ML′H_4_L type was detected. Conversely, ML′L and ML′L(OH)x types were formed only in the Cu(II)/DA/Ado and Cu(II)/DA/ADP systems. As shown by the distribution (Figure 12a,c), ternary species with Ado and ADP (as a second ligand) occurred in the whole pH range (binary Cu(II)/DA complexes were not observed). In contrast, in the systems with AMP and ATP, ternary forms were formed up to a pH of about 9.0; above this pH, only Cu(II)/DA complexes occurred (Figure 12b,d). This indicates a lower tendency of AMP and ATP to form the mixed species with dopamine at higher pH values. 

#### 2.2.1. Molecular Complexes ML′-L

Cu(AMP)H_4_(DA) species was observed in strongly acidic pH (Figure 12). As evidenced by ^31^P NMR analysis, the signal from P_α_ was shifted by −1.950 ppm, in relation to the free AMP as well as VIS spectra (λ_max_ = 800 nm), which proves that in this species, only the oxygen atom from the phosphate moiety of partially protonated AMP was engaged in coordination with Cu(II). 

In all ternary systems, the complexes of ML′H_3_L type started forming at a pH of about 3.0. Their overall stability constants (log*β*) as well as equilibrium constants that were calculated according to log*K_e_* = log*β*_ML′H3L_ − log*β*_ML′_ − log*β*_H3L_ are shown in Table 6. The log*K_e_* values for Cu(Ado)H_3_(DA), Cu(AMP)H_3_(DA), Cu(ADP)H_3_(DA), and Cu(ATP)H_3_(DA) forms were 3.78, 3.87, 3.43, and 2.90, respectively, and are characteristic for molecular complexes [57,58,59]. Moreover, they were significantly lower than for the Cu(DA) and Cu(DA)_2_ species (log*K_e_* = 16.53 and 7.47, respectively), which indicates a different mode of interaction of DA in the binary and ternary ML′-L complexes. Hence, we can conclude that fully protonated dopamine (H_3_DA, as positive centers) is engaged in non-covalent interactions with the anchoring CuL′ complex. This type of interaction is confirmed by the analysis of the distribution of species in these systems (Figure 12). The pH range of the ML′H_3_L occurrence overlapped with this for CuL′ complexes as well as for fully protonated dopamine. 

**Figure 13:**
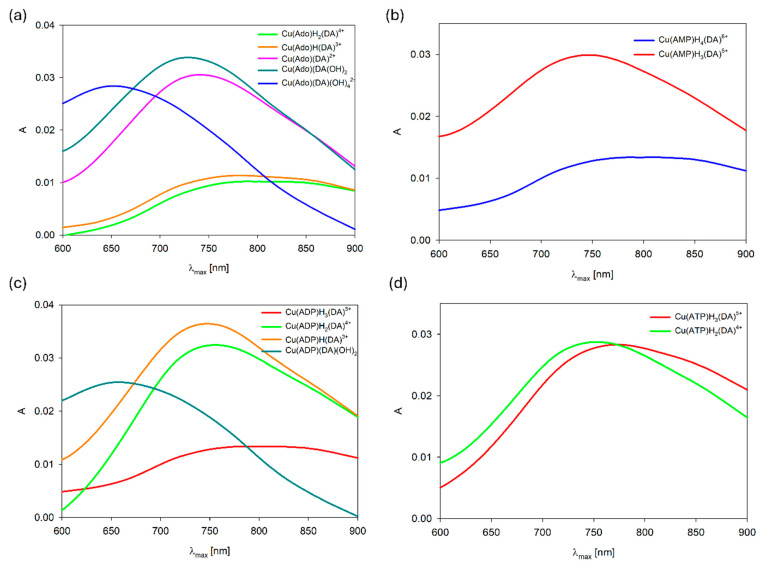
VIS spectra of the (**a**) Cu(II)/DA/Ado, (**b**) Cu(II)/DA/AMP, (**c**) Cu(II)/DA/ADP, and (**d**) Cu(II)/DA/ATP systems; C_Cu_^2+^ = 1 × 10^−3^ M, C_L=L′_ = 2 × 10^−3^ M.

Analysis of VIS (Figure 13, Table 7) and EPR spectroscopic data (Figure S3, Table 7) suggest, just like for similar systems [60,61,62], that in these molecular complexes, in the coordination to copper(II) ions, a nitrogen atom as well as oxygen atoms are involved. For example, for Cu(ATP)H_3_(DA) at pH 5.0, λ_max_ = 760 nm, g_||_ = 2.371, A_||_ = 142×10^−4^ cm^−1^, and for Cu(ATP) binary species (pH 5.0) λ_max_~750 nm, g_||_ = 2.371, A_||_ = 145×10^−4^ cm^−1^, which indicates on the same mode of interaction a nucleotide with Cu^2+^ ions, i.e., a {1N,1O} type chromophore [44]. Conversely, non-coordinated phosphate groups (P_α_ and P_β_) from ATP [44] are involved in the non-covalent interaction with H_3_DA. 

This mode of interaction is also confirmed by ^13^C NMR and ^31^P NMR studies. For example, for Cu(AMP)H_3_(DA) and Cu(ADP)H_3_(DA), significant shift signals attributed to C(2) and C(6) were −0.291, −0.253 ppm and −0.462 ppm (C(6) signal disappeared), respectively. Conversely, the ^31^P NMR spectra for Cu(AMP)H_3_(DA) showed the change in position of signals from P_α_ phosphorus atoms (relative to the free ligand) equal to −0.548 ppm, and for Cu(ADP)H_3_(DA) from P_α_, with P_β_ equal to −0.393 and 2.741 ppm. This proves that in these species, the oxygen atoms from P_α_ (for AMP) and P_β_ (for ADP) as well as N(1) or N(7) atoms are involved in the coordination. The complexes occur as a mixture of isomers (coordination dichotomy) with the chromophores {N(1),1O} and {N(7),1O} [44]. In turn, for DA, changes in the position of the signals from the carbon atoms C1, C5, and C6 for these complexes with AMP and ADP were −0.015, −0.143, and −0.146, and −0.043, −0.227, and −0.237 ppm, respectively. This indicates that the H_3_DA is engaged in a non-covalent interaction with non-coordinated negative centers of the nucleotide molecule.

#### 2.2.2. ML′H_2_L Type Complexes

With pH increasing and deprotonation of the first -OH group of dopamine (para position), a negative center is obtained, which becomes a potential site of interaction with metal ions. The involvement of this group in the coordination is suggested by the values of equilibrium constants of these species, higher than for the ML′H_3_L forms. For example, for the Cu(AMP)H_2_(DA), log*K_e_* = log*β*_Cu(AMP)H2(DA)_ − log*β*_Cu(AMP)_ − log*β*_H2DA_ = 6.80, while for the Cu(AMP)H_3_(DA), log*K_e_* = log*β*_Cu(AMP)H3(DA)_ − log*β*_Cu(AMP)_ − log*β*_H3DA_ = 3.87 (Table 6). Moreover, log*K_e_* values of the CuL′H_2_L species were lower than for Cu(DA) ({1N,1O} chromophore). This indicates a lower number of donor centers from DA included in the coordination (only the oxygen atom from the first deprotonated -OH group), e.g., in Cu(ADP)H_2_(DA) log*K_e_* = 7.95 (Table 6) and Cu(DA) log*K_e_* = 16.53 (Table 1). What is interesting is that Cu(Ado)H_2_(DA) and Cu(ADP)H_2_(DA) had higher values of the equilibrium constants (log*K_e_* = 9.57 and 7.95, respectively) in comparison to Cu(AMP)H_2_(DA) and Cu(ATP)H_2_(DA) (log*K_e_* = 6.80 and 5.22, respectively) (Table 6). A similar tendency was observed also in the Cu(II)/Ser-P/AMP or ADP or ATP systems [51]. It might be explained by the differences in the rigidity of nucleoside and nucleotide molecules. Ado and ADP (the symmetrical phosphate chain) seem to be more rigid than AMP or ATP because one or three phosphate groups may provide greater flexibility in interactions with metal ions.

The positions of the d-d maximum band as well as EPR parameters (Table 7, Figure 13) for CuL′H_2_L complexes correspond to the formation of the {1N,1O} for nucleosides and {1N,2O} chromophore for nucleotides [5,60,63]. The controversial problem of the reliability of NMR measurements of substances with paramagnetic ions had been discussed earlier, and the general conclusion was that it is possible to draw information on the mode of coordination based on changes in the signal positions [62,64,65,66], because significant changes in the chemical shifts of some atoms were observed only in the pH ranges in which the formation of complexes took place, as evidenced by the potentiometric data. To minimize the effect of the NMR signal broadening, the spectra were recorded at low concentrations of Cu(II) ions [67]. The analysis of ^13^C NMR spectra indicates unimportant changes in the position of signals corresponding to the carbon atoms C1, located close to the donor nitrogen atoms from dopamine, for these type species with Ado, ADP, and ATP, whose values are only 0.009, 0.028, and 0.110 ppm (this is small in relation to other carbon atoms shifts) respectively, and exclude this nitrogen atom from interactions (Figures S4 and S5). On the other hand, the changes in the position of the signals from the carbon atom in the vicinity of the deprotonated -OH group of the DA were, respectively, 0.069, −0.156, and −0.246 ppm for complexes with Ado, ADP, and ATP and confirm the participation of this group in the coordination with Cu(II) ions (Figures S4 and S5). Conversely, significant shifts signals attributed to C(2), C(6), and C(8) carbon atoms were, for Ado, −2.103, −1.437, and 0.718; for ADP, −0.225, −0.589, and −0.106; and for ATP, −0.142, −0.086, and 0.092 ppm (for all these complexes, the C(5) signal disappeared). This may indicate that the complexes occurred as a mixture of isomers (coordination dichotomy) with chromophores {N(1)/N(7)}. Although the N(1) atom in the adenosine ring is characterized by a much greater basicity compound to N(7), the latter is the preferred site of metalation [68,69]. Simultaneous coordination by the N(1) and N(7) and chelate ring formation is not possible for steric reasons [36]. The participation of the phosphate groups in the coordination of Cu(II) ions was confirmed by VIS and ^31^P NMR spectra analysis. The λ_max_ = 770 nm for Cu(Ado)H_2_(DA) was shifted, e.g., to the λ_max_ = 748 nm for Cu(ATP)H_2_(DA) (Figure 12a,d), as well as, e.g., for Cu(ADP)H_2_(DA), the signals P_β_ and P_α_ were shifted by 0.277 and 0.715 ppm, respectively (Figure S6). In Cu(ADP)H_2_(DA), similarly to the Cu(Ser-P)H_2_(ADP) system [51], P_α_ was incorporated in coordination with the copper(II) ion. Hence, the shifts for phosphate not engaged in the coordination may result from the participation of these groups in weak non-covalent interactions with protonated OH and -NH_3_^+^ groups of dopamine (H_2_DA). 

#### 2.2.3. ML′HL Type Complexes

In all the investigated systems, with the deprotonation of the –NH_3_^+^ group from the dopamine molecule, the CuL′HL complexes formed. As described above, due to the lack of the value log*β* for H(DA), it was not possible to calculate the log*K_e_* of these monoprotonated species. At pH 5.1, in which the Cu(Ado)H(DA) complex dominated, the λ_max_ value was close to 770 nm, while the values of parameters obtained from the EPR investigation were g_||_ = 2.345, A_||_ = 155 × 10^−4^ cm^−1^ (Table 7). Analysis of the above data indicates the presence of a {1N,1O} chromophore [60,61]. The possibility of deciding which nitrogen atom of the ligand molecules is involved in the coordination is given by the ^13^C NMR spectrum. Changes in the location of signals from carbon atoms C(2), C(6), C(5), and C(8) from Ado, related to the free ligand, were −0.437, −0.387, −0.097, and 0.006 ppm, respectively, while from the C1 carbon atom located in the vicinity of the -NH_2_ group of dopamine, it was −0.004 ppm. This clearly indicates that the N(1) nitrogen atom from Ado was engaged in metalation in the Cu(Ado)H(DA) compound. In turn, signal shifts from carbon atoms close to the deprotonated -OH group from this ligand were equal to −0.072 ppm (bigger compared to the other shifts for DA) and proved the involvement of the oxygen atom in the interaction with copper(II) ions (Figure S7). At physiological pH, monoprotonated complexes with nucleotides were formed. 

Because of the pH where the Cu(AMP)H(DA) and Cu(ATP)H(DA) formed, the mixture of other forms also occurred at high concentrations, but VIS spectra were not taken. For the Cu(ADP)H(DA) species, the position of the absorption band λ_max_ = 740 nm as well as EPR data g_||_ = 2.382, A_||_ = 121 × 10^−4^ cm^−1^ point to the coordination with the {1N,2O} type chromophore (Table 7) [61,62]. This was confirmed by NMR studies. The signals of P_α_ and P_β_ phosphate groups from ADP were shifted by −0.253 ppm and −0.445 ppm, respectively. Conversely, changes in the location of signals from carbon atoms from this ligand were C(2) −0.160 ppm, C(6) −0.082 ppm, and C(8) −0.168 ppm, and the C(5) signal disappeared. The shifts from the C1, C5, and C6 carbon atoms of dopamine were −0.090, −0.140, and −0.276 ppm. Thus, in Cu(ADP)H(DA), a nitrogen atom as well an oxygen atom from the P_β_ group of ADP and an oxygen atom from one of the deprotonated -OH groups of dopamine were involved in the coordination.

The EPR parameters for Cu(AMP)H(DA) and Cu(ATP)H(DA) were g_||_ = 2.347, A_||_ = 154 × 10^−4^ cm^−1^ and g_||_ = 2.319, A_||_ = 177 × 10^−4^ cm^−1^, respectively, indicating a similar mode of interaction in both species, i.e., {1N, 2O} type of chromophore. In addition, in the EPR spectra for Cu(ATP)H(DA), a second center appeared with g_||_ = 2.250, A_||_ = 203 × 10^−4^ cm^−1^ of binary CuH_2_(DA)_2_ species ({2N} chromophore), which was observed at a significant concentration at this pH (Table 2 and Table 7). 

The Cu(AMP)H(DA) species occurred in the pH range in which binary CuH(DA), Cu(DA), and CuH_2_(DA)_2_ complexes were also observed (Figure 12b), and therefore it is difficult to determine, based on the analysis of ^13^C NMR spectra, which center of DA takes part in the coordination. On the other hand, it is possible to determine which donor centers of the AMP molecule are involved in coordination in this form. In the ^13^C NMR spectra, the shifts for nucleotides from C(2), C(6), C(8), and C(5) were −0.101, 0.012, and 0.311 ppm (C(5) signal disappeared), respectively, while in the ^31^P NMR spectrum, a change in the position of the signal of the phosphorus atom from –PO_4_ was observed, equal to −0.123 ppm, in relation to free AMP. This clearly indicates coordination with Cu(II) ions of the nitrogen atom and the oxygen atom with AMP (Figure S8).

#### 2.2.4. ML′L and ML′L(OH)_x_ Type Complex

The mixed ML′L as well as ML′L(OH)x species are formed only in the ternary Cu(II)/DA/Ado and Cu(II)/DA/ADP systems (Figure 12a,c). The values of the overall stability and equilibrium constants are provided in Table 6. The Cu(Ado)(DA) complex dominated at pH 6.3, binding 70% of Cu(II) ions. Analysis of VIS spectra and EPR data (Table 7), λ_max_ = 740 nm, g_||_ = 2.329, A_||_ = 158 × 10^−4^ cm^−1^, indicate coordination with the {1N,1O} type chromophore. Which of the nitrogen atoms from the ligand molecules directly participated in metalation was determined based on ^13^C NMR spectroscopy. The changes in the chemical shifts of the C(8) and C(5) carbon atoms, which were located close to the donor N(7) nitrogen atoms of Ado, relative to their position in the spectra of free ligand, were 0.112 and −0.150 ppm, respectively. However, the shifts from the atoms located close to the N(1) nitrogen atom to Ado were only −0.057 ppm for carbon C(2) and −0.027 ppm for carbon C(6) atoms. These changes indicate the involvement of the N(7) nitrogen atom in the copper(II) coordination. On the other hand, the shift of the signal from the C1 carbon atom for dopamine was only −0.019 ppm and excluded the participation in the metalation of the nitrogen atom from the amino group of this ligand. Moreover, the shifts of C5 and C6 carbon atoms were −0.090 and −0.084 ppm, respectively (not big, but much bigger compared to the C1 shift for DA), indicating that in this complex, the center of interaction of DA was an oxygen atom from one of the deprotonated -OH groups. In contrast, Cu(ADP)(DA) occurred in the pH range of the dominance of Cu(ADP)H(DA) and Cu(ADP)(DA)(OH)_2_ forms (Figure 12c), and therefore it was not possible to take spectral investigations and determine the mode of coordination in this species. 

In both of these systems, the hydroxocomplexes were formed from pH > 6.0. In the Cu(II)/DA/Ado system, computer analysis of potentiometric data indicate the formation of Cu(Ado)(DA)(OH)_2_ as well as Cu(Ado)(DA)(OH)_4_ species. These complexes occur in the pH range in which, in the Cu(II)/Ado system, Cu(Ado)(OH)_2_ as well as Cu(Ado)(OH)_3_ species are observed [42], but which were not detected in this ternary system. This can also explain the very high concentration of these ternary hydroxocomplexes (Figure 12a). Conversely, the decrease in the log*K_e_* values of these mixed forms (Table 6), with the increase in the number of -OH groups involved in coordination, suggests that further hydroxo groups bond with less energy, which may be caused by steric hindrance. 

As was described earlier, the position of the d-d transition band assigned to Cu(Ado)(OH)_2_ species (pH~7.0, λ_max_ = 750 nm) as well as ^13^C NMR data points to the metal binding by the N(7) nitrogen atom from the purine ring of adenosine and two -OH groups from water molecules [42]. Conversely, for the Cu(Ado)(DA)(OH)_2_ complex at pH 7.0, λ_max_ = 725 nm, which suggests similar mode of interaction of Ado and involving the coordination the next donor center, i.e., an oxygen atom from the deprotonated -OH group of dopamine. We excluded the involvement of a nitrogen atom from DA in the metalation, because the coordination of nitrogen atom causes shifts close to about 50 nm [70,71]. In turn, the position of the absorption band, λ_max_ = 650 nm at pH 8.5 (Figure 13a), at which Cu(Ado)(DA)(OH)_4_ dominates, indicates that in the metalation, two nitrogen atoms and two further -OH groups from water molecules are already involved (in this pH, dopamine interacts only by the –NH_2_ group, as was observed for the binary system). 

**Figure 14:**
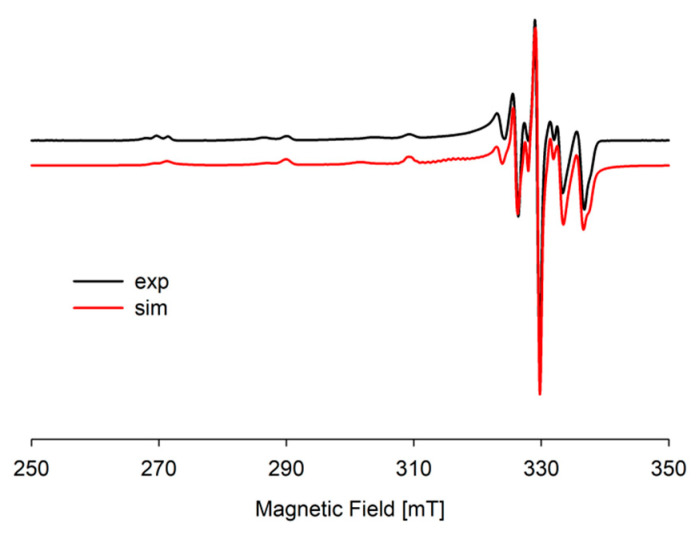
Experimental and simulated EPR spectra for the Cu(II)/DA/ADP system at pH 8.0; C_Cu_^2+^ = 1 × 10^−3^ M, C_L=L′_ = 2 × 10^−3^ M.

In the Cu(II)/DA/ADP system, at pH 8.0 for Cu(ADP)(DA)(OH)_2_, λ_max_ = 657 nm. In addition, the EPR data indicate the occurrence of two interaction centers, first with g_||_ = 2.292, A_||_ = 162 × 10^−4^ cm^−1^ and second with g_||_ = 2.250, A_||_ = 205 × 10^−4^ cm^−1^ (Figure 14, Table 7). These parameters may suggest the existence of a mixture of two isomers of this hydroxocomplex with {2N,4O} type chromophore. Similarly as in the Cu(ADP)(DA) complex, in Cu(ADP)(DA)(OH) species, spectroscopic studies could not be carried out because it occurs at high concentrations of other species, i.e., Cu(ADP)H(DA) and Cu(ADP)(DA)(OH)_2_.

## 3. Materials and Methods

### 3.1. Chemicals 

Dopamine hydrochloride (DA)—C_8_H_11_NO_2_ × HCl (purity 98%), adenosine (Ado)—C_10_H_13_N_5_O_4_ (purity 99%), adenosine 5′-monophosphate monohydrate (AMP)—C_10_H_14_N_5_O_7_P×H_2_O (purity 97%), adenosine 5′-diphosphate sodium salt (ADP)—C_10_H_14_N_5_O_10_P_2_Na (purity 97%), adenosine 5′-triphosphate disodium salt trihydrate (ATP)—C_10_H_14_N_5_O_13_P_3_Na_2_×3H_2_O (purity 99%), and copper(II) nitrate(V) trihydrate—Cu(NO_3_)_2_×3H_2_O (purity 99%) were purchased from Sigma-Aldrich (Steinheim am Albuch Baden-Württemberg, Germany) and were used without further purification. The concentrations of copper(II), Cl^−^, and Na^+^ ions were determined by the method of inductively coupled plasma optical emission spectrometry (ICP OES). All solutions and experiments were prepared using demineralized carbonate-free water. D_2_O, NaOD, and DCl were purchased from the Sigma-Aldrich (Steinheim am Albuch Baden-Württemberg, Germany).

### 3.2. Potentiometric Studies

Potentiometric measurements were carried out using Metrohm 702 SM Titrino equipped with an autoburette with a combined glass electrode—Metrohm 6.0233.100 (Metrohm AG, Herisau, Switzerland) calibrated in terms of hydrogen ions concentration [6] with initial calibration using a phthalate buffer (pH = 4.002) and borax buffer (pH = 9.225). Potentiometric titrations were carried out for the copper(II) concentration of 1 × 10^−3^ M, at the M:L ratios of 1:1, 1:2, and 1:4 for binary systems and M:L′:L ratios of 1:1:1 and 1:2:2 for all ternary systems (where L = DA; L′ = Ado, AMP, ADP, or ATP). All measurements were carried out in the atmosphere of neutral gas (Argon 5N) (Linde Gaz, Krakow, Poland), at the constant ionic strength of KNO_3_ (*µ* = 0.1 M), temperature of 20 ± 1 °C, in the pH range from 2.5 to 11.0, using as a titrant CO_2_-free NaOH solution (~0.20 M). The initial volume of the sample was 30 mL. In the case of the binary system in the molar ratio M:L = 1:1 and 1:2 as well as ternary system M:L:L′ = 1:1:1 from pH~6, precipitate formation was observed, so these systems were not considered in the calculations. The model choice and determination of stability constants (log*β*) of complexes formed in the systems studied were selected with the aid of the HYPERQUAD 2008 program [6] only for systems in which no precipitate appeared over the entire pH range. For each system, at least six titrations were made, and 150–250 points from each titration curve were used for computer analysis.

### 3.3. Spectroscopic Measurements

The mode of coordination was established based on spectroscopic studies. The spectra were recorded at pH where the concentration of complex forms is the highest and in sufficient excess in relation to other forms which are also observed at this pH.

#### 3.3.1. VIS Spectroscopy 

Samples for visible spectroscopy studies were prepared in H_2_O, and the metal concentration was 1 × 10^−5^ M (for UV–VIS spectra of the DA and Cu(II)/DA system depending on the pH) and 1 × 10^−3^ M (for the rest of the UV–VIS measurements) at the ratios M:L = 1:4 and M:L:L′ = 1:2:2. Spectra were recorded at room temperature on an Evolution 300 UV/VIS Thermo Fisher Scientific spectrophotometer (Thermo Electron Scientific Instruments LLC, Madison, WI, USA) using a Plastibrand PMMA (Brand, Wertheim, Germany) cell with 1 cm path length. 

#### 3.3.2. EPR Spectroscopy 

EPR studies were carried out at −196 °C, using glass capillary tubes (volume 130 μm^3^). The concentration of Cu(II) was 1 × 10^−3^ M in a water/ethylene glycol mixture (3:1), and the metal/ligand ratios were 1:4 for binary and 1:2:2 for ternary systems. The spectra were recorded on Bruker Elexsys E580 (Bruker, Billerica, MA, USA) and SE/X 2547 Radiopan (Radiopan, Poznan, Poland) spectrometers. The all-experimental spectra were simulated using the computer program EPRsim32 (version 0.3 alfa, Krakow, Poland) [72].

#### 3.3.3. IR Spectroscopy 

Samples for IR measurements were prepared by dissolving Cu(II) and dopamine in D_2_O and adjusting the pH by the addition of NaOD and DCl. The value of the pH was corrected according to the formula pD = pH_readings_ + 0.4. The metal concentration in the samples was 1 × 10^−3^ M, and Cu(II) to dopamine concentrations were 1:4. IR spectra were recorded on an IR Spirit Fourier Transform Infrared Spectrophotometer spectrometer (Shimadzu) (Shimadzu, Kyoto, Japan).

#### 3.3.4. NMR Spectroscopy 

The samples for ^13^C NMR and ^31^P NMR studies were prepared by dissolving Cu(II) and ligands in D_2_O at a metal/ligand1 ratio of 1:100 as well as metal/ligand1/ligand2 ratio of 1:50:50. The concentration of the metal was 1 × 10^−3^ M. The pH was adjusted just like for IR studies. The ^13^C NMR and ^31^P NMR spectra were recorded on an NMR Varian 400MHz spectrometer (Varian, Palo Alto, CA, USA) using dioxane or phosphoric acid, respectively, as internal standard. To minimize the broadening of the NMR signals related to the paramagnetic character of Cu(II) ions, the spectra were taken at low metal concentrations and huge excess of ligands [73]. NMR studies were carried out for the samples, in which precipitate formation was not observed and for complexes in the pH of their dominance. Each time, the spectrum of the complex was compared with the spectra of the free ligands (at the same pH and concentration). Only shifts relative to the free ligand are reported.

### 3.4. Quantum-Chemical Calculations

**Figure 15:**
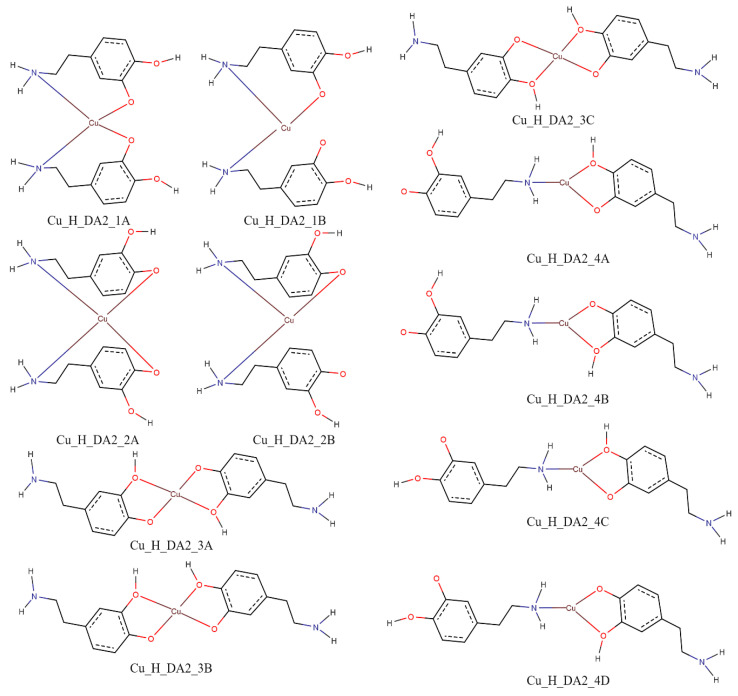
Schemes of a possible interaction between two protonated molecules of dopamine with a copper(II) ion; red—oxygen atoms, blue—nitrogen atoms and brown—copper(II) ions.

**Figure 16:**
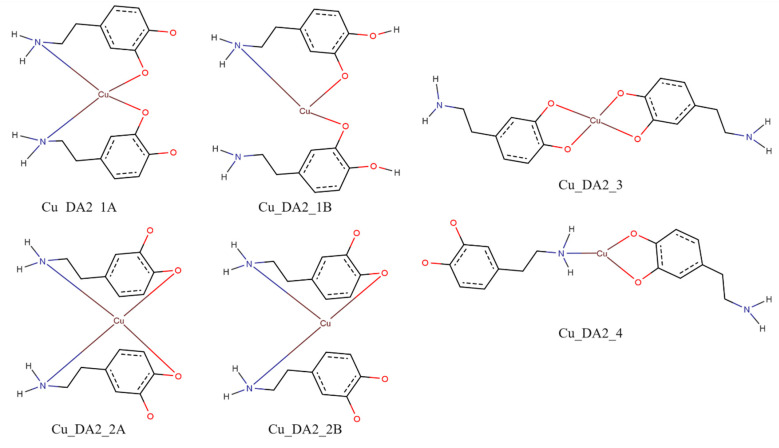
Schemes of a possible interaction between two molecules of dopamine with a copper(II) ion; red—oxygen atoms, blue—nitrogen atoms and brown—copper(II) ions.

The interaction energy between compounds in an aqueous solution was calculated for six possible schemes of interaction for complexes consisting of dopamine or protonated dopamine with a copper(II) ion (Figure 11), for eleven possible schemes of interaction for complexes consisting of two molecules of protonated dopamine and a copper(II) ion (Figure 15), and for six possible schemes of interaction for complexes consisting of two molecules of dopamine and a copper(II) ion (Figure 16). Energy calculations were carried out with the M06 [74] method and in the SDD [75] basis set, which is recommended for non-covalent interactions [76,77,78,79,80]. To take into account the effect of aqueous solvent, we used the Polarizable Continuum Model (PCM) [81].

Figure 11 shows schemes of a possible interaction between a single molecule of dopamine or protonated dopamine and a copper(II) ion. Figure 15 shows the investigated possible interaction schemes of two molecules of protonated dopamine with a copper(II) ion. 

In Figure 16, we depicted possible schemes of interaction of two molecules of dopamine with a copper(II) ion.

## 4. Conclusions

This study aimed to determine, in aqueous solution, the mode of coordination of copper(II) ions in the binary Cu(II)/DA as well as ternary Cu(II)/DA/Ado (AMP, ADP, ATP) systems and deciphering the impact of nucleosides and nucleotides on copper ion and dopamine coordination dynamics. These systems are rather sophisticated, and their equilibrium state is determined by a competition between many intermolecular interactions such as hydrogen bonds, van der Waals interactions, coordination interactions, and electrostatic interactions between charged atoms and molecular groups. Potentiometric measurements enabled the identification of various types of Cu(II) complexes and to determine their protonation state in the dynamical equilibrium state at a constant temperature.

Based on spectral and computational studies (combined with equilibrium studies), it was established that in the Cu(II)/DA system, depending on the pH value, the active centers of the ligand involved in the coordination with copper(II) ions changed from nitrogen and oxygen atoms in the CuH(DA)^3+^ and Cu(DA)^2+^ complex, via nitrogen atoms in the CuH_2_(DA)_2_^4+^ species, to oxygen atoms at strongly alkaline pH in the Cu(DA)_2_^2+^ compound. The introduction of Ado or adenosine phosphates into this system changes the mode of interaction of dopamine, and the oxygen atoms from the deprotonated -OH groups of this ligand are the main center of coordination. In contrast, the nitrogen atom from DA takes part in interaction only in the hydroxocomplexes, which are formed in the Cu(II)/DA/Ado and Cu(II)/DA/ADP systems.

In all ternary systems two types of mixed complexes were found, i.e., molecular complexes ML′-L (with complex–ligand non-covalent interaction) and protonated ML′H_x_L. The ML′L and ML′L(OH)_x_ type species were formed only in the Cu(II)/DA/Ado or ADP systems. In complexes that occurred up to physiological pH, a N(1)/N(7) coordination dichotomy was observed, i.e., interactions with copper(II) ions involved either the N(1) or (N(7) nitrogen atom from L′.

In the Cu(II)/DA/AMP or ATP systems, mixed forms were formed up to a pH of around 9.0; above this pH, only Cu(II)/DA complexes occurred. This indicates a low tendency of AMP and ATP to form the mixed species with dopamine at higher pH values. In contrast to systems with AMP and ATP, ternary species with Ado and ADP occurred in the whole pH range in high concentration; moreover, binary complexes of Cu(II) ions with dopamine did not form in a detectable concentration.

## Figures and Tables

**Table 1:** Protonation constants of DA, overall stability constants (log*β*), and equilibrium constants (log*K_e_*) for complexes formed in the Cu(II)/DA binary system, as well as the literature data.

Systems	Species	log*β*	log*K_e_*	Ref.
DA	H_3_(DA)^3+^	32.20 ± 0.07	8.82	32.40 [32], 31.71 [39], 31.79 [31]
	H_2_(DA)^2+^	23.38 ± 0.06	-	23.51 [32], 22.65 [39], 22.94 [31]
	H(DA)^+^	not determined	-	
Cu(II)/DA	CuH(DA)^3+^	23.93 ± 0.01	-	24.22 [32], 23.29 [39]
	Cu(DA)^2+^	16.53 ± 0.05	16.53	16.60 [32], 16.01 [39]
	CuH_2_(DA)^24+^	45.37 ± 0.02	-	45.83 [32], 44.26 [39]
	CuH(DA)^23+^	34.89 ± 0.05	-	35.66 [32]
	Cu(DA)^22+^	24.00 ± 0.04	7.47	24.78 [32], 23.47 [39]

**Table 2:** Visible and EPR spectral data and chromophore type for the Cu(II)/DA complexes.

Species	pH	λ_max_ [nm]	g_||_	g_⊥_	A_||_ [10^−4^ cm^−1^]	A_⊥_ [10^−4^ cm^−1^]	Type of Chromophore
CuH(DA)^3+^	6.0	740	2.318	2.060	171	<5	{1N,1O}
CuH_2_(DA)_2_^4+^	8.0	658	2.292 2.250	2.048 2.051	156 203	18.44 31.90	{2N}

**Table 3:** Calculated energies, sum of monomers, and interaction energy of possible dopamine complexes with copper(II) ion in water.

Interaction Scheme of Dopamine–Cu Complex	Energy [Hartree]	The Sum of Monomers [Hartree]	Interaction Energy [kcal/mol]
Cu_H_DA1	−712.867571	−712.596618	−170.0
Cu_H_DA2	−712.882092	−712.550343	−208.2
Cu_DA1	−712.457026	−712.057465	−250.7
Cu_DA2	−712.453956	−712.091191	−227.6
Cu_DA3	−712.349837	−711.909721	−276.2

**Table 4:** Calculated energies, sum of monomers, and interaction energy of possible complexes of two protonated dopamine molecules with copper (II) in water.

Interaction Scheme of Two Molecules of Protonated Dopamine–Cu Complex	Energy [Hartree]	The Sum of Monomers [Hartree]	Interaction Energy [kcal/mol]
Cu_H_DA2_1_A	−1228.788705	−1228.383236	−254.4
Cu_H_DA2_1_B	−1228.788130	−1228.394962	−246.7
Cu_H_DA2_2_A1	−1228.807307	−1228.239747	−356.1
Cu_H_DA2_2_A2	−1228.787186	−1228.380653	−255.1
Cu_H_DA2_2_B	−1228.760212	−1228.372483	−243.3
Cu_H_DA2_3_A	−1228.771797	−1228.367769	−253.5
Cu_H_DA2_3_B	−1228.772301	−1228.368866	−253.2
Cu_H_DA2_3_C	−1228.772810	−1228.369879	−252.8
Cu_H_DA2_4_A	−1228.769173	−1228.375143	−247.3
Cu_H_DA2_4_B	−1228.771144	−1228.374988	−248.6
Cu_H_DA2_4_C	−1228.776615	−1228.397126	−238.1
Cu_H_DA2_4_D	−1228.774482	−1228.386332	−243.6

**Table 5:** Calculated energies, sum of monomers, and interaction energy of possible complexes of the two dopamine molecules with copper(II) in water.

Interaction Scheme of Two Molecules of Dopamine–Cu Complex	Energy [Hartree]	The Sum of Monomers [Hartree]	Interaction Energy [kcal/mol]
Cu_DA2_1_A	−1227.826653	−1227.351948	−297.9
Cu_DA2_1_B	−1227.813830	−1227.355787	−287.4
Cu_DA2_2_A	−1227.826654	−1227.351935	−297.9
Cu_DA2_2_B	−1227.797549	−1227.350360	−280.6
Cu_DA2_3	−1227.865274	−1227.356821	−319.1
Cu_DA2_4	−1227.836650	−1227.364976	−296.0

**Table 6:** Overall stability constants (log*β*) and equilibrium constants (log*K_e_*) for complexes formed in Cu(II)/DA/Ado, Cu(II)/DA/AMP, Cu(II)/DA/ADP, and Cu(II)/DA/ATP ternary systems.

Systems	Species	Reaction	log*β*	log*K_e_*
Cu(II)/DA/Ado	Cu(Ado)H_3_(DA)^5+^	Cu(Ado)^2+^ + H_3_(DA)^3+^ ⇌ Cu(Ado)H_3_(DA)^5+^	38.86 ± 0.06	3.78
	Cu(Ado)H_2_(DA)^4+^	Cu(Ado)^2+^ + H_2_(DA)^2+^ ⇌ Cu(Ado)H_2_(DA)^4+^	35.83 ± 0.03	9.57
	Cu(Ado)H(DA)^3+^	Cu(Ado)^2+^ + H(DA)^+^ ⇌ Cu(Ado)H(DA)^3+^	31.19 ± 0.03	-
	Cu(Ado)(DA)^2+^	Cu(Ado)^2+^ + DA ⇌ Cu(Ado)(DA)^2+^	25.51 ± 0.03	22.63
	Cu(Ado)(DA)(OH)_2_	Cu(Ado)(DA)^2+^ + 2H_2_O ⇌ Cu(Ado)(DA)(OH)_2_ + 2H+	12.09 ± 0.03	14.58
	Cu(Ado)(DA)(OH)_4_^2−^	Cu(Ado)(DA)(OH)_2_ + 2H_2_O ⇌ Cu(Ado)(DA)(OH)_4_^2−^+ 2H+	−2.77 ± 0.04	13.14
Cu(II)/DA/AMP	Cu(AMP)H_4_(DA)^6+^	Cu^2+^ + H(AMP)^+^ + H_3_(DA)^3+^ ⇌ Cu(AMP)H_4_(DA)^6+^	44.29 ± 0.04	-
	Cu(AMP)H_3_(DA)^5+^	Cu(AMP)^2+^ + H_3_(DA)^3+^ ⇌ Cu(AMP)H_3_(DA)^5+^	39.09 ± 0.04	3.87
	Cu(AMP)H_2_(DA)^4+^	Cu(AMP)^2+^ + H_2_(DA)^2+^ ⇌ Cu(AMP)H_2_(DA)^4+^	33.20 ± 0.06	6.80
	Cu(AMP)H(DA)^3+^	Cu(AMP)^2+^ + H(DA)^+^ ⇌ Cu(AMP)H(DA)^3+^	26.97 ± 0.04	-
Cu(II)/DA/ADP	Cu(ADP)H_3_(DA)^5+^	Cu(ADP)^2+^ + H_3_(DA)^3+^ ⇌ Cu(ADP)H_3_(DA)^5+^	42.62 ± 0.04	3.43
	Cu(ADP)H_2_(DA)^4+^	Cu(ADP)^2+^ + H_2_(DA)^2+^ ⇌ Cu(ADP)H_2_(DA)^4+^	38.32 ± 0.03	7.95
	Cu(ADP)H(DA)^3+^	Cu(ADP)^2+^ + H(DA)^+^ ⇌ Cu(ADP)H(DA)^3+^	32.39 ± 0.04	-
	Cu(ADP)(DA)^2+^	Cu(ADP)^2+^ + DA ⇌ Cu(ADP)(DA)^2+^	25.05 ± 0.04	18.06
	Cu(Ado)(DA)(OH)^+^	Cu(ADP)(DA)^2+^ + H_2_O ⇌ Cu(ADP)(DA)(OH)+ + H^+^	18.07 ± 0.04	7.02
	Cu(Ado)(DA)(OH)_2_	Cu(ADP)(DA)(OH)^+^ + H_2_O ⇌ Cu(ADP)(DA)(OH)_2_ + H^+^	10.77 ± 0.04	6.70
Cu(II)/DA/ATP	Cu(ATP)H_3_(DA)^5+^	Cu(ATP)^2+^ + H_3_(DA)^3+^ ⇌ Cu(ATP)H_3_(DA)^5+^	41.73 ± 0.03	2.90
	Cu(ATP)H_2_(DA)^4+^	Cu(ATP)^2+^ + H_2_(DA)^2+^ ⇌ Cu(AMP)H_2_(DA)^4+^	35.23 ± 0.03	5.22
	Cu(ATP)H(DA)^3+^	Cu(ATP)^2+^ + H(DA)^+^ ⇌ Cu(AMP)H(DA)^3+^	27.56 ± 0.07	-

log*β* for: Cu(OH)_2_ = −13.13 [54]; H(Ado)^+^ = 3.92 ± 0.01; Cu(Ado)^2+^ = 2.88 ± 0.15; Cu(Ado)(OH)_2_ = −11.41 ± 0.08; Cu(Ado)(OH)_3_^−^ = −20.92 ± 0.14 [42]; H_2_(AMP)^2+^ = 10.45 ± 0.02; H(AMP)^+^ = 6.43 ± 0.02; Cu(AMP)^2+^ = 3.02 ± 0.08; Cu(AMP)(OH)^+^ = −3.82 ± 0.05 [43]; H_2_(ADP)^2+^ = 10.63 ± 0.02; H(ADP)^+^ = 6.55 ± 0.01; CuH(ADP)^3+^ = 10.84 ± 0.03; Cu(ADP)^2+^ = 6.99 ± 0.06; Cu(ADP)(OH)^+^ = −1.03 ± 0.02 [36]; H_2_(ATP)^2+^ = 10.88 ± 0.02; H(ATP)^+^ = 6.50 ± 0.01; CuH(ATP)^3+^ = 10.53 ± 0.03; Cu(ATP)^2+^ = 6.63 ± 0.02; Cu(ATP)(OH)^+^ = −1.25 ± 0.02 [44].

**Table 7:** VIS and EPR spectroscopic parameters for complexes in the Cu(II)/DA/nucleoside (or nucleotide) ternary system.

Systems	Species	pH	λ_max_ [nm]	g_||_	g_⊥_	A_||_ [10^−4^ cm^−1^]	A_⊥_ [10^−4^ cm^−1^]
Cu(II)/DA/Ado	Cu(Ado)H_2_(DA)^4+^	4.0	770	2.394	2.080	141	5.98
	Cu(Ado)H(DA)^3+^	5.1	770	2.345	2.075	155	<5
	Cu(Ado)(DA)^2+^	6.3	740	2.329	2.071	158	<5
	Cu(Ado)(DA)(OH)_2_	7.0	725	-	-	-	-
	Cu(Ado)(DA)(OH)_4_^2−^	8.5	650	-	-	-	-
Cu(II)/DA/AMP	Cu(AMP)H_4_(DA)^6+^	4.3	795	-	-	-	-
	Cu(AMP)H_3_(DA)^5+^	5.5	745	2.345	2.072	156	<5
	Cu(AMP)H_2_(DA)^4+^	6.3	-	2.316	2.069	173	<5
	Cu(AMP)H(DA)^3+^	6.8	-	2.347	2.070	154	<5
Cu(II)/DA/ADP	Cu(ADP)H_3_(DA)^5+^	3.8	770	2.392	2.072	130	6.06
	Cu(ADP)H_2_(DA)^4+^	5.1	754	2.342	2.071	168	5.41
	Cu(ADP)H(DA)^3+^	6.5	740	2.382	2.053	121	<5
	Cu(ADP)(DA)(OH)_2_	8.0	657	2.292	2.048	162	17.69
				2.250	2.052	205	33.13
Cu(II)/DA/ATP	Cu(ATP)H_3_(DA)^5+^	5.0	760	2.371	2.074	142	<5
	Cu(ATP)H_2_(DA)^4+^	7.0	748	2.362	2.076	149	<5
	Cu(ATP)H(DA)^3+^	7.4	-	2.319	2.047	177	15.59
				2.250	2.052	203	30.30

## Data Availability

Data are contained within the article and Supplementary Materials.

## Supplementary Materials

The following supporting information can be downloaded at https://www.mdpi.com/article/10.3390/ijms25179137/s1.
