# Supplementary material for: Deciphering the Impact of Nucleosides and Nucleotides on Copper Ion and Dopamine Coordination Dynamics
Source: Int J Mol Sci. 2024 Aug 23;25(17):9137. doi: 10.3390/ijms25179137 (PMC11394690; doi:10.3390/ijms25179137)
Supplement: Supplementary file 1 [file ijms-25-09137-s001.zip › ijms-3173773-supplementary.pdf]

Supporting Information

Deciphering the Impact of Nucleosides and Nucleotides on Copper Ion and Dopamine Coordination Dynamics

Patrycja Sadowska [1], Wojciech Jankowski [1], Romualda Bregier-Jarzębowska [1], Piotr Pietrzyk [2] and Renata Jastrząb [1,*]

[1] Faculty of Chemistry, Adam Mickiewicz University in Poznań, Uniwersytetu Poznańskiego 8, 61-614 Poznań, Poland; patrycja.sadowska@amu.edu.pl (P.S.); wojciech.jankowski@amu.edu.pl (W.J.); bregier@amu.edu.pl (R.B.-J.)
[2] Faculty of Chemistry, Jagiellonian University, Gronostajowa 2, 30-387 Kraków, Poland; piotr.pietrzyk@uj.edu.pl
* Correspondence: renatad@amu.edu.pl

Table of contents

Table/Figure	Title	Page
**Table S1.**	Atomic coordinates of optimized structures of protonated dopamine complexes with copper(II) ion from interaction schemes Cu_H_DA1 and Cu_H_DA2.	**3**
**Table S2.**	Atomic coordinates of optimized structures of dopamine complexes with copper(II) ion from interaction schemes Cu_DA1, Cu_DA2 and Cu_DA3.	**4**
**Table S3.**	Atomic coordinates of optimized structures of complexes consisted of two protonated molecules of dopamine and copper(II) ion from interaction schemes Cu_H_DA2_1_A, Cu_H_DA2_1_B and Cu_H_DA2_2_A1.	**5-6**
**Table S4.**	Atomic coordinates of optimized structures of complexes consisted of two protonated molecules of dopamine and copper(II) ion from interaction schemes Cu_H_DA2_2_A2, Cu_H_DA2_2_B and Cu_H_DA2_3_A.	**6-7**
**Table S5.**	Atomic coordinates of optimized structures of complexes consisted of two protonated molecules of dopamine and copper(II) ion from interaction schemes Cu_H_DA2_3_B, Cu_H_DA2_3_C and Cu_H_DA2_4_A.	**7-8**
**Table S6.**	Atomic coordinates of optimized structures of complexes consisted of two protonated molecules of dopamine and copper(II) ion from interaction schemes Cu_H_DA2_4_B, Cu_H_DA2_4_C and Cu_H_DA2_4_D.	**8-9**
**Table S7.**	Atomic coordinates of optimized structures of complexes consisted of two molecules of dopamine and copper(II) ion from interaction schemes Cu_DA2_1_A, Cu_DA2_1_B and Cu_DA2_2_A.	**9-10**
**Table S8.**	Atomic coordinates of optimized structures of complexes consisted of two molecules of dopamine and copper(II) ion from interaction schemes Cu_DA2_2_B, Cu_DA2_3 and Cu_DA2_4.	**10-11**
**Figure S1.**	Optimized structures of obtained complexes consisted of two protonated molecules of dopamine and copper(II) ion.	**12**
**Figure S2.**	Optimized structures of obtained complexes consisted of two molecules of dopamine and copper(II) ion.	**13**
**Figure S3.**	Experimental and simulated EPR spectra for the ML′H_3L systems $C_{Cu^{2+}}=1\times10^{-3}$ M, $C_{L=L'}=2\times10^{-3}$M.	**13**
**Figure S4.**	^{13}C NMR spectra of Cu(II)/Ado/DA system at pH 4.0; $C_{Cu^{2+}}=1\times10^{-3}$ M, $C_{L=L'}=5\times10^{-2}$ M.	**14**
**Figure S5.**	^{13}C NMR spectra of Cu(II)/ADP/DA system at pH 5.1; $C_{Cu^{2+}}=1\times10^{-3}$ M, $C_{L=L'}=5\times10^{-2}$ M.	**14**
**Figure S6.**	^{31}P NMR spectra of Cu(II)/ADP/DA system at pH 5.5; $C_{Cu^{2+}}=1\times10^{-3}$ M, $C_{L=L'}=5\times10^{-2}$ M.	**15**
**Figure S7.**	^{13}C NMR spectra of Cu(II)/Ado/DA system at pH 5.1; $C_{Cu^{2+}}=1\times10^{-3}$ M, $C_{L=L'}=5\times10^{-2}$ M.	**15**
**Figure S8.**	^{31}P NMR spectra of Cu(II)/AMP/DA system at pH 6.8; $C_{Cu^{2+}}=1\times10^{-3}$ M, $C_{L=L'}=5\times10^{-2}$ M.	**15**
**Figure S9.**	^{31}P NMR spectra of AMP at pH 6.8; $C_{Cu^{2+}}=1\times10^{-3}$ M, $C_L=5\times10^{-2}$ M.	**16**
**Figure S10.**	^{31}P NMR spectra of ADP at pH 5.1; $C_{Cu^{2+}}=1\times10^{-3}$ M, $C_L=5\times10^{-2}$ M.	**16**

Table S1. Atomic coordinates of optimized structures of protonated dopamine complexes with copper(II) ion from interaction schemes Cu_H_DA1 and Cu_H_DA2.

Dopamine - Cu(II) complexes atomic coordinates						
	Cu_H_DA1			Cu_H_DA2		
Atom	x	y	z	x	y	z
C	-0.86613	0.58723	0.03574	-1.26988	1.13872	-0.49525
C	0.26379	-0.23025	0.33531	0.01086	1.64400	-0.73860
C	1.52713	0.31064	0.48486	1.01582	1.44924	0.22642
C	1.70131	1.73062	0.32337	0.65525	1.12314	1.57652
C	0.63287	2.56981	0.03719	-0.57257	0.54944	1.81796
C	-0.65351	2.02482	-0.10917	-1.43513	0.28364	0.68299
H	0.10221	-1.30064	0.44998	0.26891	2.01927	-1.72622
C	2.73053	-0.54734	0.72173	2.41139	1.13220	-0.22100
H	2.69859	2.14779	0.43649	1.40606	1.17631	2.35900
H	0.78088	3.63951	-0.07400	-0.84342	0.12333	2.77758
O	-1.75461	2.75948	-0.38396	-2.18069	-0.80841	0.62774
O	-2.07674	0.12735	-0.11017	-2.30556	1.16114	-1.36698
H	2.45113	-1.49844	1.18986	3.17951	1.56852	0.42671
H	3.44985	-0.04109	1.37804	2.59268	1.46988	-1.24666
C	3.44709	-0.85879	-0.63086	2.58019	-0.41210	-0.12279
H	2.73202	-1.36789	-1.28982	3.55084	-0.67741	-0.55385
N	4.64339	-1.67757	-0.49511	1.50841	-1.22301	-0.82808
H	3.71653	0.08661	-1.11877	2.57709	-0.72062	0.92649
H	4.47909	-2.61334	-0.13509	1.40594	-0.88840	-1.79370
H	5.42153	-1.22112	-0.02809	1.84446	-2.19024	-0.88820
Cu	-2.71716	-1.60085	-0.00375	-0.30693	-1.35314	-0.09420
H	-1.58569	3.72121	-0.47013	-2.12567	1.64996	-2.19654

Table S2. Atomic coordinates of optimized structures of dopamine complexes with copper(II) ion from interaction schemes Cu_DA1, Cu_DA2 and Cu_DA3.

Dopamine - Cu(II) complexes atomic coordinates									
	Cu_DA1			Cu_DA2			Cu_DA3		
Atom	x	y	z	x	y	z	x	y	z
C	-1.23918	-0.53966	0.56803	0.58950	-0.47875	-0.22640	-1.24801	-0.99121	-0.87447
C	-0.20706	0.26433	1.14854	-0.76731	-0.82703	-0.47140	-2.27938	0.08631	-0.60461
C	0.07637	1.51249	0.61351	-1.76302	0.13887	-0.50011	-1.65165	1.16991	-0.01222
C	-0.92416	2.15147	-0.19212	-1.42051	1.51891	-0.27295	-1.57058	0.93870	1.45392
C	-2.11871	1.51605	-0.47364	-0.11892	1.91063	-0.03408	-1.05518	-0.33497	1.63190
C	-2.35185	0.12388	-0.13155	0.92984	0.94341	-0.00041	-0.67802	-1.22849	0.49794
H	0.50314	-0.22393	1.81915	-1.00436	-1.87531	-0.63853	-3.08328	-0.33734	0.00800
C	1.52171	1.93040	0.58381	-3.20759	-0.22837	-0.68370	-0.58983	1.97290	-0.74144
H	-0.72640	3.14755	-0.58222	-2.21620	2.26057	-0.30107	-1.63572	1.66819	2.25605
H	-2.89861	2.01154	-1.04679	0.14313	2.95139	0.13016	-0.71194	-0.66793	2.61056
O	-3.40649	-0.51640	-0.50115	2.18132	1.27431	0.22204	0.37936	-2.06343	0.66284
O	-0.99240	-1.85274	0.44576	1.55202	-1.37413	-0.19518	-0.85109	-1.55441	-1.92207
H	2.03867	1.67615	1.51826	-3.30475	-1.20981	-1.16487	-1.03416	2.95575	-0.95637
H	1.63641	3.00780	0.41970	-3.72085	0.50969	-1.31560	-0.43391	1.51830	-1.73444
C	2.22740	1.22349	-0.60736	-3.93929	-0.27922	0.68739	0.76121	2.23393	-0.04738
H	1.63894	1.37516	-1.51895	-3.43624	-1.01834	1.32423	1.12599	3.22769	-0.32891
N	2.43126	-0.26830	-0.45209	-5.35984	-0.61282	0.60179	1.82794	1.22775	-0.41710
H	3.20960	1.68626	-0.75565	-3.83429	0.69359	1.18527	0.65505	2.21403	1.04166
Cu	0.85865	-1.37527	-0.14979	-5.54513	-1.54452	0.23924	1.96585	1.23346	-1.43315
H	2.89980	-0.61666	-1.29399	-5.92268	0.08854	0.12738	2.71638	1.51106	0.00755
H	3.06277	-0.44440	0.33683	3.26474	-0.41235	0.17229	1.42354	-0.55370	0.12761

Table S3. Atomic coordinates of optimized structures of complexes consisted of two protonated molecules of dopamine and copper(II) ion from interaction schemes Cu_H_DA2_1_A, Cu_H_DA2_1_B and Cu_H_DA2_2_A1.

Two molecules of protonated dopamine - Cu complex atomic coordinates									
	Cu_H_DA2_1_A			Cu_H_DA2_1_B			Cu_H_DA2_2_A1		
Atom	x	y	z	x	y	z	x	y	z
C	2.04300	0.42700	-1.47700	-2.52500	-1.55300	-0.68300	2.37400	-0.95900	0.10400
C	1.55000	-0.89100	-1.29900	-2.52100	-0.21600	-1.14900	1.55300	-1.98700	-0.37700
C	2.06300	-1.75500	-0.30700	-3.65800	0.61500	-1.02400	0.55100	-2.55700	0.42400
C	3.11100	-1.32200	0.52500	-4.83800	0.09800	-0.46700	0.40000	-2.09200	1.74500
C	3.64600	-0.02100	0.36800	-4.88900	-1.24300	-0.02400	1.20700	-1.05500	2.23400
C	3.11100	0.82300	-0.59700	-3.75300	-2.03500	-0.12400	2.18200	-0.46700	1.41400
H	0.75300	-1.23700	-1.95900	-1.61900	0.16000	-1.63800	1.71900	-2.34300	-1.39300
C	1.43700	-3.11900	-0.11800	-3.56300	2.08400	-1.35900	-0.34300	-3.64000	-0.13100
H	3.51400	-1.99000	1.28400	-5.71300	0.73700	-0.36600	-0.35400	-2.54000	2.39000
H	4.45900	0.32900	0.99800	-5.79500	-1.65600	0.41100	1.08500	-0.67700	3.24500
O	3.55300	2.13500	-0.76900	-3.72200	-3.34900	0.34100	2.94100	0.57700	1.90600
O	1.58500	1.32100	-2.34000	-1.46000	-2.35900	-0.64200	3.39500	-0.43800	-0.67700
H	2.20300	-3.87000	0.10800	-4.50600	2.44500	-1.78800	-0.44900	-4.46100	0.58900
H	0.93800	-3.43500	-1.04500	-2.77600	2.27100	-2.10300	0.10800	-4.06600	-1.03800
C	0.41500	-3.15900	1.02800	-3.28400	2.93200	-0.10800	-1.75300	-3.14300	-0.47000
H	-0.02300	-4.16200	1.09500	-3.41800	3.99600	-0.33700	-2.32000	-3.95800	-0.93400
N	-0.67000	-2.14300	0.81800	-1.89500	2.71500	0.41600	-1.69600	-1.96200	-1.39100
H	0.90500	-2.93300	1.98200	-3.98700	2.66100	0.68700	-2.28200	-2.84100	0.44100
H	-1.07700	-2.25400	-0.12100	-1.21800	3.06300	-0.27200	-0.95700	-2.09200	-2.08900
H	-1.42100	-2.28100	1.50000	-1.76200	3.26400	1.27100	-2.58000	-1.86400	-1.89700
Cu	0.00400	-0.33700	1.01600	-1.32600	0.91600	0.83100	-1.47000	-0.20300	-0.47400
H	2.99300	2.49400	-1.50700	-2.79200	-3.65500	0.18400	3.02400	0.14900	-1.40800
C	-2.36300	-0.25200	-1.22400	5.20700	0.86300	-0.22600	1.31400	2.14200	-0.38700
C	-1.38600	0.80100	-1.04100	3.77100	0.90700	-0.16800	0.25400	2.57800	0.48100
C	-1.58200	1.82900	-0.13300	3.02400	-0.25500	-0.18000	-1.07900	2.42800	0.15800
C	-2.80400	1.88200	0.60200	3.69200	-1.52400	-0.25200	-1.44100	1.83700	-1.10900
C	-3.79400	0.90300	0.45300	5.08200	-1.62700	-0.31200	-0.45700	1.32400	-1.96400
C	-3.59000	-0.16100	-0.43000	5.83900	-0.45600	-0.29900	0.94400	1.41800	-1.63000
H	-0.48100	0.77500	-1.65100	3.29600	1.88400	-0.11500	0.54600	3.00300	1.44100
C	-0.48700	2.82000	0.16100	1.52100	-0.23000	-0.07300	-2.17000	2.70100	1.16000
H	-2.96900	2.70200	1.29700	3.08700	-2.42800	-0.26900	-2.48000	1.88600	-1.43900
H	-4.71400	0.96600	1.02700	5.57800	-2.58900	-0.37100	-0.71500	0.92600	-2.94200
O	-4.48700	-1.17300	-0.61200	7.19600	-0.49100	-0.35700	1.86100	0.88100	-2.37800
O	-2.14400	-1.25200	-2.00300	5.97400	1.89900	-0.22000	2.56400	2.32000	-0.08500
H	-0.86900	3.84700	0.12100	1.07100	-0.96500	-0.75600	-2.81700	3.52700	0.83800
H	0.31500	2.72800	-0.58700	1.13200	0.76000	-0.34900	-1.72000	2.99600	2.11500
C	0.08800	2.59700	1.56800	1.07500	-0.56500	1.35700	-3.06200	1.45900	1.36600
H	0.76800	3.41900	1.82300	1.53200	-1.51300	1.67500	-3.61900	1.56400	2.30300
N	0.81800	1.29500	1.67200	-0.41200	-0.67300	1.41100	-2.24100	0.20600	1.37000
H	-0.71800	2.58700	2.30900	1.40700	0.21800	2.04900	-3.79100	1.36700	0.55400
H	1.70500	1.34400	1.14800	-0.71800	-1.42900	0.75300	-1.44400	0.29100	2.01200

H	1.05700	1.11500	2.65200	-0.71500	-0.91500	2.35800	-2.80400	-0.59200	1.67400
H	-5.29200	-1.08700	-0.06200	7.55000	0.43300	-0.33700	3.10200	1.25600	1.18900

Table S4. Atomic coordinates of optimized structures of complexes consisted of two protonated molecules of dopamine and copper(II) ion from interaction schemes Cu_H_DA2_2_A2, Cu_H_DA2_2_B and Cu_H_DA2_3_A.

Two molecules of protonated dopamine - Cu complex atomic coordinates									
	Cu_H_DA2_2_A2			Cu_H_DA2_2_B			Cu_H_DA2_3_A		
Atom	x	y	z	x	y	z	x	y	z
C	2.99200	0.10500	0.05000	1.29000	-2.02600	0.90200	-2.80100	0.19500	-0.33200
C	2.33800	1.14000	0.70300	2.07600	-1.06700	1.54400	-4.09800	0.63400	-0.55400
C	1.58100	2.07200	-0.04900	3.25600	-0.59100	0.94900	-5.15000	-0.30300	-0.49700
C	1.55700	1.93200	-1.44700	3.65300	-1.09900	-0.32600	-4.84000	-1.64600	-0.21200
C	2.23700	0.88900	-2.10300	2.89200	-2.04500	-0.97900	-3.52000	-2.07100	0.00900
C	2.96500	-0.08800	-1.37200	1.66300	-2.55900	-0.40800	-2.46500	-1.14600	-0.04800
H	2.42600	1.24000	1.78600	1.77400	-0.68500	2.51800	-4.30500	1.68100	-0.77000
C	0.74100	3.11600	0.64400	4.11500	0.42100	1.66200	-6.58200	0.14200	-0.65500
H	0.98300	2.64800	-2.03600	4.57900	-0.74300	-0.77300	-5.64900	-2.37300	-0.16700
H	2.19600	0.80000	-3.18700	3.18600	-2.44600	-1.94400	-3.29100	-3.11100	0.22300
O	3.59400	-1.14900	-1.85800	0.93700	-3.42600	-1.01900	-1.15800	-1.46900	0.15000
O	3.67600	-0.89000	0.75700	0.13900	-2.52900	1.43400	-1.65800	1.02700	-0.36700
H	0.73500	4.05200	0.07100	5.12400	0.01100	1.80400	-7.18900	-0.65700	-1.10400
H	1.14900	3.35200	1.63800	3.71100	0.62100	2.66200	-6.65000	1.01700	-1.31700
C	-0.72300	2.68000	0.79800	4.26900	1.74400	0.90500	-7.20500	0.51700	0.71200
H	-1.33200	3.51800	1.15900	4.91600	2.41400	1.48100	-7.13900	-0.35300	1.38000
N	-0.86400	1.52700	1.75000	2.93300	2.39500	0.68700	-8.60000	0.95900	0.64900
H	-1.11800	2.36200	-0.17400	4.73200	1.58900	-0.07400	-6.60100	1.31300	1.16900
H	-0.53600	1.81800	2.67800	2.41800	2.43900	1.57400	-9.24400	0.23600	0.33400
H	-1.85400	1.28200	1.85000	3.06500	3.36000	0.36900	-8.73200	1.81900	0.12200
Cu	0.09400	-0.11100	1.36600	1.85000	1.54200	-0.63300	-0.00000	-0.00000	0.00100
H	4.03100	-1.49100	0.04400	-0.09600	-2.14000	2.30100	2.80100	-0.19600	0.33200
C	-3.49400	-0.58600	-0.27400	-4.54800	1.01400	0.45200	4.09800	-0.63500	0.55300
C	-2.67000	-1.66800	0.03000	-3.18000	1.06600	0.19100	5.15000	0.30200	0.49600
C	-1.37600	-1.76500	-0.51400	-2.47000	-0.07800	-0.24200	4.84000	1.64500	0.21200
C	-0.90900	-0.74100	-1.39700	-3.19200	-1.27300	-0.39400	3.51900	2.07000	-0.00800
C	-1.71200	0.33100	-1.71900	-4.56900	-1.32600	-0.13200	2.46500	1.14500	0.04900
C	-3.04400	0.48000	-1.17100	-5.33200	-0.19400	0.30500	4.30500	-1.68200	0.76900
H	-3.02700	-2.44900	0.69900	-2.64500	2.00900	0.33300	6.58200	-0.14300	0.65400
C	-0.52100	-2.97100	-0.22300	-0.99400	-0.00500	-0.54100	5.64900	2.37200	0.16600
H	0.08900	-0.81000	-1.82700	-2.66800	-2.17100	-0.72200	3.29100	3.11000	-0.22200
H	-1.37000	1.11000	-2.39600	-5.11400	-2.26200	-0.25500	1.15700	1.46900	-0.14800
O	-3.78200	1.49500	-1.44700	-6.61800	-0.24300	0.55300	1.65800	-1.02700	0.36800
O	-4.75400	-0.43400	0.23100	-5.24800	2.15400	0.88200	6.65000	-1.02000	1.31300

H	-0.48800	-3.61000	-1.11700	-0.61000	-1.02100	-0.71300	7.18900	0.65500	1.10400
H	-0.98100	-3.57100	0.57200	-0.43300	0.41100	0.31700	7.20500	-0.51400	-0.71500
C	0.92100	-2.63700	0.15800	-0.68700	0.85200	-1.77100	6.60200	-1.30900	-1.17400
H	1.48700	-3.56700	0.28700	-1.23400	0.46700	-2.64100	8.60100	-0.95600	-0.65200
N	0.97300	-1.83300	1.41900	0.78900	0.84600	-2.05600	7.14000	0.35700	-1.38100
H	1.42400	-2.05900	-0.62700	-0.99300	1.89100	-1.60700	8.73300	-1.81800	-0.12800
H	0.59100	-2.37800	2.19800	1.09700	-0.11500	-2.24800	9.24400	-0.23400	-0.33400
H	1.96500	-1.62100	1.61900	0.99200	1.39900	-2.89400	-1.78500	1.97200	-0.56100
H	-5.02500	-1.16100	0.82800	-4.65400	2.92500	0.95700	1.78500	-1.97300	0.56000

Table S5. Atomic coordinates of optimized structures of complexes consisted of two protonated molecules of dopamine and copper(II) ion from interaction schemes Cu_H_DA2_3_B, Cu_H_DA2_3_C and Cu_H_DA2_4_A.

Two molecules of protonated dopamine - Cu complex atomic coordinates									
	Cu_H_DA2_3_B			Cu_H_DA2_3_C			Cu_H_DA2_4_A		
Atom	x	y	z	x	y	z	x	y	z
C	-2.70900	0.15800	-0.34400	-2.70100	-0.09700	0.30700	-5.35400	-1.20400	-0.24900
C	-3.90700	0.82100	-0.56300	-4.01000	-0.54500	0.54200	-4.97000	0.12600	-0.09400
C	-5.11500	0.12300	-0.35500	-5.09800	0.34700	0.48400	-3.76500	0.45400	0.55700
C	-5.05300	-1.21600	0.07400	-4.87400	1.70300	0.18300	-2.93300	-0.58800	1.06400
C	-3.82900	-1.86900	0.29000	-3.57100	2.17900	-0.05600	-3.29400	-1.91100	0.92100
C	-2.62100	-1.18400	0.08100	-2.52400	1.27200	0.01100	-4.52100	-2.29600	0.25800
H	-3.92100	1.85900	-0.89300	-4.17000	-1.59600	0.77400	-5.61100	0.92200	-0.47100
C	-6.44000	0.82600	-0.51000	-6.50400	-0.16900	0.66500	-3.35100	1.89700	0.68400
H	-5.98300	-1.75800	0.23700	-5.71600	2.39000	0.13800	-2.00900	-0.32000	1.57100
H	-3.79300	-2.90400	0.61600	-3.39000	3.22600	-0.28400	-2.67200	-2.71600	1.30200
O	-1.39100	-1.73900	0.26200	-1.16200	1.59300	-0.19900	-4.86400	-3.52700	0.12500
O	-1.43100	0.73700	-0.52200	-1.60700	-0.90300	0.35300	-6.51100	-1.58600	-0.86500
H	-7.22200	0.11500	-0.81200	-7.15800	0.61500	1.07100	-2.51500	1.99000	1.38900
H	-6.38400	1.60300	-1.28600	-6.52300	-1.01400	1.36700	-4.18300	2.49600	1.07500
C	-6.87700	1.49600	0.81600	-7.09500	-0.64700	-0.68500	-2.94100	2.50400	-0.66500
H	-6.93700	0.72600	1.59800	-7.08000	0.19100	-1.39500	-3.80000	2.52600	-1.34800
N	-8.16000	2.20200	0.75100	-8.46000	-1.17300	-0.60300	-1.81900	1.73400	-1.29100
H	-6.09800	2.20400	1.12900	-6.44100	-1.42500	-1.10100	-2.60000	3.53100	-0.51300
H	-8.95200	1.59200	0.56600	-9.14800	-0.47900	-0.32000	-2.14700	0.80200	-1.56800
H	-8.15500	2.99800	0.11700	-8.53900	-2.01600	-0.03900	-1.51100	2.20900	-2.14500
Cu	0.01700	-0.55700	-0.11100	-0.00000	0.00000	0.00000	-0.29100	1.49500	-0.17300
H	2.65600	0.07400	-0.31300	2.70100	0.09700	-0.30700	2.41800	-0.01900	-0.37800
C	3.85700	0.76100	-0.54100	4.00900	0.54500	-0.54300	3.58800	-0.74200	-0.61300
C	5.09900	0.12400	-0.34800	5.09800	-0.34700	-0.48500	4.61500	-0.78200	0.35200
C	5.13700	-1.21500	0.08400	4.87400	-1.70300	-0.18300	4.42500	-0.06900	1.54800
C	3.94700	-1.92700	0.31800	3.57100	-2.17800	0.05700	3.24600	0.65600	1.78100
C	2.74300	-1.26900	0.11500	2.52400	-1.27200	-0.01000	2.20800	0.70400	0.82700

C	3.81500	1.79600	-0.87300	4.17000	1.59600	-0.77500	3.71000	-1.28500	-1.55200
H	6.38000	0.90000	-0.52600	6.50300	0.16900	-0.66600	5.90900	-1.50100	0.06500
C	6.09700	-1.70400	0.23300	5.71600	-2.39000	-0.13800	5.20600	-0.08500	2.30600
H	3.96800	-2.96300	0.64500	3.39000	-3.22500	0.28500	3.10000	1.20200	2.71000
H	1.46500	-1.84800	0.30000	1.16200	-1.59300	0.20000	1.07500	1.40300	1.06200
O	1.42600	0.62600	-0.48600	1.60700	0.90300	-0.35300	1.37300	0.04200	-1.30800
O	6.27000	1.66200	-1.31100	6.52300	1.01500	-1.36800	5.73600	-2.38600	-0.56500
H	7.20000	0.23300	-0.82400	7.15700	-0.61500	-1.07300	6.37300	-1.84900	0.99900
H	6.78400	1.61300	0.78900	7.09500	0.64600	0.68400	6.91500	-0.57800	-0.66400
C	5.96600	2.27900	1.09500	6.44200	1.42400	1.10100	6.45600	-0.22100	-1.59600
H	8.02200	2.39100	0.70600	8.46100	1.17100	0.60100	8.20400	-1.20300	-0.98200
N	6.89200	0.85900	1.58100	7.08100	-0.19300	1.39300	7.09700	0.30800	-0.04100
H	7.97000	3.17400	0.05700	8.54000	2.01500	0.03800	8.12700	-1.98100	-1.63300
H	8.84900	1.82600	0.52900	9.14900	0.47700	0.31900	8.73900	-1.47000	-0.15900
H	-1.38400	1.66100	-0.82300	0.93700	-2.51200	0.42600	-7.04800	-0.83500	-1.19000
H	1.42100	-2.77100	0.60700	-0.93700	2.51300	-0.42500	1.55300	-0.47100	-2.11900

Table S6. Atomic coordinates of optimized structures of complexes consisted of two protonated molecules of dopamine and copper(II) ion from interaction schemes Cu_H_DA2_4_B, Cu_H_DA2_4_C and Cu_H_DA2_4_D.

Two molecules of protonated dopamine - Cu complex atomic coordinates									
	Cu_H_DA2_4_B			Cu_H_DA2_4_C			Cu_H_DA2_4_D		
Atom	x	y	z	x	y	z	x	y	z
C	4.00600	1.92100	0.21400	4.99400	-0.81000	0.11100	5.40100	0.88200	-0.50200
C	3.95100	0.67300	0.82900	4.57400	0.55100	-0.08600	4.93400	-0.40400	-0.06200
C	2.71300	0.09800	1.17700	3.41000	0.84500	-0.77300	3.76900	-0.52500	0.67400
C	1.50700	0.80800	0.89900	2.61700	-0.22700	-1.30500	3.02100	0.65300	1.00800
C	1.53800	2.04600	0.29200	2.97700	-1.56900	-1.14200	3.42800	1.92900	0.60500
C	2.78500	2.67300	-0.08700	4.14400	-1.86700	-0.44100	4.59800	2.05200	-0.14100
H	4.87400	0.14000	1.05200	5.21000	1.33400	0.32000	5.53200	-1.27400	-0.32400
C	2.66600	-1.26300	1.82200	2.95000	2.27300	-0.95500	3.26100	-1.88100	1.10600
H	0.55700	0.35500	1.17400	1.72300	0.01900	-1.87500	2.11700	0.54500	1.60400
H	0.62600	2.59600	0.07600	2.37600	-2.37100	-1.55600	2.85600	2.81300	0.86500
O	2.82400	3.82500	-0.65500	4.54500	-3.14900	-0.25100	5.04600	3.26400	-0.55900
O	5.17300	2.53000	-0.14500	6.06300	-1.16300	0.73800	6.47500	1.07700	-1.18900
H	1.63500	-1.50400	2.10900	2.04600	2.29600	-1.57700	2.43200	-1.76100	1.81500
H	3.27100	-1.26600	2.73800	3.71600	2.85300	-1.48500	4.05400	-2.43600	1.62100
C	3.20600	-2.37500	0.91300	2.66600	2.98700	0.37300	2.79000	-2.73500	-0.07800
H	4.28600	-2.25400	0.75400	3.60000	3.19000	0.91100	3.64400	-3.02400	-0.70400
N	2.50600	-2.39700	-0.41300	1.75500	2.18200	1.25500	1.78200	-2.00600	-0.91600
H	3.04200	-3.34200	1.39300	2.17900	3.94400	0.17300	2.31800	-3.64700	0.29500
H	2.82100	-1.60600	-0.98700	2.28500	1.42600	1.70200	2.24900	-1.26300	-1.44800
H	2.75100	-3.25200	-0.91800	1.38900	2.77500	2.00600	1.36900	-2.64900	-1.59700

Cu	0.60600	-2.23800	-0.32500	0.30600	1.35400	0.35000	0.37100	-1.17500	0.06300
H	-1.92000	-1.07600	-0.50200	-2.05900	-0.14100	-0.08400	-2.51800	-0.17700	-0.50600
C	-3.26200	-0.88800	-0.11000	-3.36600	0.36500	-0.04400	-3.78800	0.23100	-0.91400
C	-4.00700	0.23900	-0.50000	-4.43000	-0.39500	0.48400	-4.65900	0.87600	-0.01300
C	-3.39700	1.21800	-1.30400	-4.16800	-1.68700	0.97500	-4.21100	1.08900	1.30200
C	-2.06600	1.05500	-1.72300	-2.86500	-2.21500	0.94200	-2.93200	0.67800	1.70800
C	-1.34400	-0.07300	-1.33000	-1.82800	-1.45000	0.41400	-2.04800	0.03400	0.81800
C	-3.71600	-1.65200	0.52200	-3.54700	1.36900	-0.42700	-4.11000	0.05200	-1.94100
H	-5.41300	0.43600	0.01200	-5.83800	0.14700	0.44500	-6.05800	1.25000	-0.43600
C	-3.96100	2.09700	-1.60800	-4.98200	-2.28000	1.38400	-4.86900	1.58500	2.01400
H	-1.59100	1.80000	-2.36000	-2.64900	-3.21200	1.31800	-2.58600	0.85000	2.72500
H	-0.02400	-0.30700	-1.74300	-0.53000	-1.95600	0.37400	-0.81500	-0.35500	1.21400
O	-1.22600	-2.16600	-0.10700	-1.00400	0.54300	-0.62300	-1.62000	-0.81600	-1.37000
O	-5.91500	-0.53100	0.15900	-5.84600	1.23000	0.63700	-6.08000	1.55500	-1.49200
H	-6.01100	1.01400	-0.70700	-6.45900	-0.32900	1.21700	-6.42900	2.09700	0.15800
H	-5.41100	1.19300	1.36200	-6.49600	-0.10400	-0.93300	-7.03700	0.06400	-0.25600
C	-4.81200	0.62400	2.08600	-5.88100	0.36800	-1.71100	-6.66900	-0.79000	-0.84000
H	-6.74000	1.43200	1.93400	-7.87100	0.39200	-1.05600	-8.42100	0.34500	-0.65200
N	-4.90700	2.16000	1.22400	-6.49000	-1.18400	-1.13700	-7.02600	-0.24600	0.79800
H	-7.23600	0.57800	2.18100	-7.94500	1.40400	-0.97700	-8.52600	0.54900	-1.64300
H	-7.32500	2.03900	1.36300	-8.52600	-0.07000	-0.42900	-8.86700	1.06300	-0.08400
H	5.97000	2.00200	0.06700	5.39300	-3.15900	0.25800	5.88700	3.14800	-1.06600
H	0.40800	0.49200	-2.10200	0.04200	-1.30100	-0.08700	-1.97800	-0.93900	-2.27000

Table S7. Atomic coordinates of optimized structures of complexes consisted of two molecules of dopamine and copper(II) ion from interaction schemes Cu_DA2_1_A, Cu_DA2_1_B and Cu_DA2_2_A.

Two molecules of dopamine - Cu complexes atomic coordinates									
	Cu_DA2_1_A			Cu_DA2_1_B			Cu_DA2_2_A		
Atom	x	y	z	x	y	z	x	y	z
C	2.33800	0.54100	-1.13900	-2.48300	-1.35400	-1.29700	-2.33700	-0.54100	-1.14000
C	1.56000	-0.67100	-1.05400	-3.10300	-0.07200	-1.33800	-1.56000	0.67200	-1.05400
C	1.91900	-1.71700	-0.21100	-4.02100	0.40700	-0.36000	-1.91900	1.71700	-0.21000
C	3.11100	-1.61500	0.57700	-4.36400	-0.43900	0.69900	-3.11200	1.61300	0.57800
C	3.90400	-0.48400	0.52600	-3.74200	-1.71300	0.80800	-3.90300	0.48200	0.52600
C	3.57000	0.64600	-0.30600	-2.74600	-2.17200	-0.09000	-3.56900	-0.64800	-0.30700
H	0.64900	-0.74700	-1.65900	-2.86500	0.56200	-2.19900	-0.64900	0.74900	-1.65900
C	1.01900	-2.90900	-0.02000	-4.44400	1.85500	-0.40000	-1.02100	2.91000	-0.01800
H	3.38400	-2.44900	1.22400	-5.07200	-0.10400	1.45900	-3.38500	2.44600	1.22600
H	4.81000	-0.40500	1.12500	-3.95700	-2.34500	1.67300	-4.80900	0.40100	1.12500
O	4.29800	1.71800	-0.31900	-1.98700	-3.23800	0.17100	-4.29600	-1.72000	-0.32100
O	1.94600	1.55100	-1.85600	-1.65400	-1.75900	-2.24100	-1.94400	-1.55000	-1.85800
H	1.60400	-3.83800	0.00400	-5.45200	1.99300	0.01300	-1.60700	3.83800	0.00700
H	0.29700	-2.98300	-0.84400	-4.46300	2.22400	-1.43700	-0.30000	2.98600	-0.84300
C	0.24600	-2.81700	1.30700	-3.49600	2.75000	0.42100	-0.24700	2.81700	1.30800

H	-0.24200	-3.77900	1.51600	-3.77300	3.80600	0.30000	0.24000	3.78000	1.51800
N	-0.77800	-1.73300	1.25500	-2.06500	2.55000	0.01400	0.77800	1.73400	1.25400
H	0.93800	-2.60300	2.13000	-3.57200	2.49200	1.48400	-0.93900	2.60200	2.13200
H	-1.52500	-1.99800	0.58400	-1.96100	2.72400	-0.99100	1.52400	2.00100	0.58200
H	-1.22300	-1.63300	2.17200	-1.46100	3.20900	0.50900	1.22500	1.63500	2.17100
Cu	-0.18000	0.07700	0.76500	-1.44900	0.72400	0.37300	0.18100	-0.07600	0.76500
C	-2.02200	-0.40100	-1.44300	4.99500	1.10900	-0.32100	2.02100	0.39900	-1.44400
C	-1.61700	0.93800	-1.17600	3.57900	0.84700	-0.38300	1.61700	-0.94000	-1.17600
C	-2.22300	1.77300	-0.19700	3.03400	-0.40600	-0.15200	2.22300	-1.77300	-0.19600
C	-3.30300	1.27000	0.53400	3.90400	-1.50100	0.16400	3.30300	-1.26900	0.53400
C	-3.71100	-0.07700	0.34000	5.27300	-1.32000	0.23500	3.71000	0.07800	0.33900
C	-3.05600	-0.96500	-0.54900	5.89400	-0.03900	0.00400	3.05500	0.96500	-0.55100
H	-0.79200	1.33600	-1.77600	2.93100	1.69000	-0.62500	0.79100	-1.33800	-1.77400
C	-1.57600	3.09300	0.14300	1.54200	-0.62500	-0.16500	1.57700	-3.09300	0.14600
H	-3.79600	1.89400	1.28200	3.46700	-2.48300	0.33900	3.79600	-1.89200	1.28200
H	-4.50900	-0.49500	0.95900	5.93600	-2.15200	0.46800	4.50800	0.49700	0.95800
O	-3.27800	-2.28200	-0.53100	7.17800	0.11500	0.07200	3.27500	2.28200	-0.53400
O	-1.44600	-1.13300	-2.38200	5.47700	2.29200	-0.53600	1.44500	1.13000	-2.38400
H	-2.31900	3.82900	0.48200	1.29600	-1.59400	-0.62400	2.31900	-3.82800	0.48600
H	-1.07500	3.51900	-0.74000	1.03800	0.15700	-0.75400	1.07500	-3.52000	-0.73700
C	-0.53500	2.94900	1.26800	0.97900	-0.60000	1.26000	0.53500	-2.94800	1.27100
H	-0.05300	3.91900	1.45600	1.43600	-1.40700	1.85100	0.05300	-3.91800	1.46000
N	0.49900	1.92500	0.91800	-0.50600	-0.74400	1.23800	-0.49900	-1.92400	0.92000
H	-1.03100	2.62600	2.19100	1.22900	0.35400	1.73900	1.03100	-2.62400	2.19400
H	0.90200	2.12900	-0.01000	-0.78700	-1.64000	0.79000	-0.90200	-2.12800	-0.00800
H	1.25800	1.93700	1.60400	-0.87200	-0.76900	2.19500	-1.25800	-1.93500	1.60500

Table S8. Atomic coordinates of optimized structures of complexes consisted of two molecules of dopamine and copper(II) ion from interaction schemes Cu_DA2_2_B, Cu_DA2_3 and Cu_DA2_4.

Two molecules of dopamine - Cu complexes atomic coordinates									
	Cu_DA2_2_B			Cu_DA2_3			Cu_DA2_4		
Atom	x	y	z	x	y	z	x	y	z
C	1.96600	-1.67500	1.45700	-2.63700	0.08700	-0.36800	5.25000	-1.29200	-0.23100
C	2.06700	-0.32500	1.96900	-3.85800	0.73600	-0.60100	4.30300	-0.73700	-1.16400
C	2.83300	0.66200	1.37500	-5.08900	0.09300	-0.33100	2.95700	-0.57600	-0.87000
C	3.60000	0.34600	0.19300	-5.07400	-1.21500	0.18000	2.47200	-0.97800	0.41800
C	3.47000	-0.91700	-0.40700	-3.85200	-1.88300	0.42300	3.32600	-1.51600	1.36400
C	2.63700	-1.96200	0.15600	-2.63300	-1.24500	0.15800	4.73400	-1.70000	1.11200
H	1.46100	-0.09100	2.84400	-3.84800	1.75300	-0.99800	4.68900	-0.44200	-2.14200
C	2.73300	2.10800	1.79200	-6.39200	0.83600	-0.50900	2.00900	0.05700	-1.86400
H	4.32500	1.06300	-0.18800	-6.01800	-1.71700	0.39100	1.41000	-0.85200	0.63700
H	4.05800	-1.17700	-1.28500	-3.83900	-2.89900	0.81500	2.95700	-1.82100	2.34200
O	2.46000	-3.08100	-0.46800	-1.41000	-1.81300	0.36600	5.52300	-2.20100	2.00800
O	1.27800	-2.58000	2.06500	-1.42000	0.64500	-0.61300	6.50000	-1.43800	-0.53400

H	3.67400	2.47300	2.22500	-7.20900	0.14000	-0.75000	0.96500	-0.15100	-1.59300
H	1.96100	2.21300	2.56500	-6.32200	1.55900	-1.33600	2.19000	-0.34000	-2.87100
C	2.39700	3.01500	0.59000	-6.77900	1.61000	0.77300	2.18700	1.58000	-1.93300
H	2.08300	4.00100	0.95300	-6.86300	0.89600	1.60400	3.18200	1.82500	-2.33200
N	1.34000	2.40600	-0.27800	-8.03100	2.37300	0.68000	2.01900	2.20000	-0.58100
H	3.28200	3.16000	-0.03700	-5.96400	2.30000	1.03000	1.43100	2.01300	-2.59600
H	0.47700	2.26200	0.25700	-8.84800	1.78600	0.52600	2.74800	1.84600	0.04800
H	1.12100	3.02300	-1.06400	-7.99300	3.12200	-0.00900	2.13000	3.21500	-0.64000
Cu	1.86400	0.56900	-1.00300	0.00100	-0.58200	-0.13200	0.31500	1.81500	0.25000
C	-4.36000	1.02400	0.10300	2.63500	0.10900	-0.34700	-2.23000	0.65700	-0.27600
C	-3.00600	0.70900	-0.20100	3.85200	0.77300	-0.55400	-3.42300	0.06000	-0.74100
C	-2.47200	-0.61000	-0.21500	5.08700	0.11400	-0.34700	-4.56300	-0.10100	0.08100
C	-3.32200	-1.67300	0.09700	5.08000	-1.22600	0.07400	-4.50900	0.35100	1.40900
C	-4.68400	-1.41600	0.41000	3.86100	-1.91000	0.28600	-3.33200	0.95900	1.90600
C	-5.26200	-0.11600	0.43100	2.63800	-1.25800	0.07900	-2.19900	1.12300	1.09600
H	-2.34500	1.55300	-0.43300	3.83700	1.81500	-0.88000	-3.45400	-0.28800	-1.77700
C	-1.01600	-0.83300	-0.55700	6.38600	0.86900	-0.49500	-5.78600	-0.81300	-0.44900
H	-2.94000	-2.69600	0.09900	6.02700	-1.74000	0.23600	-5.38000	0.23300	2.05500
H	-5.34900	-2.25000	0.65200	3.85300	-2.95100	0.60700	-3.28400	1.31600	2.93400
O	-6.53800	0.08700	0.72700	1.41900	-1.84200	0.25700	-1.05900	1.71200	1.55900
O	-4.81100	2.27000	0.09900	1.41400	0.68300	-0.53000	-1.13400	0.81700	-1.03600
H	-0.75800	-1.89800	-0.43800	6.31200	1.62600	-1.29100	-5.98100	-0.53600	-1.49600
H	-0.35500	-0.27100	0.13300	7.20500	0.18700	-0.76800	-6.67800	-0.53600	0.13400
C	-0.68100	-0.39800	-1.98600	6.77600	1.58900	0.81700	-5.62800	-2.35000	-0.38500
H	-1.26500	-0.99500	-2.70100	5.96000	2.26500	1.10600	-4.74000	-2.64100	-0.96400
N	0.78600	-0.53900	-2.25100	8.02500	2.36100	0.75000	-6.78200	-3.11100	-0.88500
H	-0.94500	0.65700	-2.13100	6.86600	0.84100	1.61700	-5.43600	-2.64400	0.65600
H	1.08200	-1.51100	-2.09700	7.98300	3.13400	0.09000	-6.96600	-2.95400	-1.87400
H	1.01300	-0.28800	-3.21600	8.84400	1.78300	0.57400	-7.62700	-2.96000	-0.33700

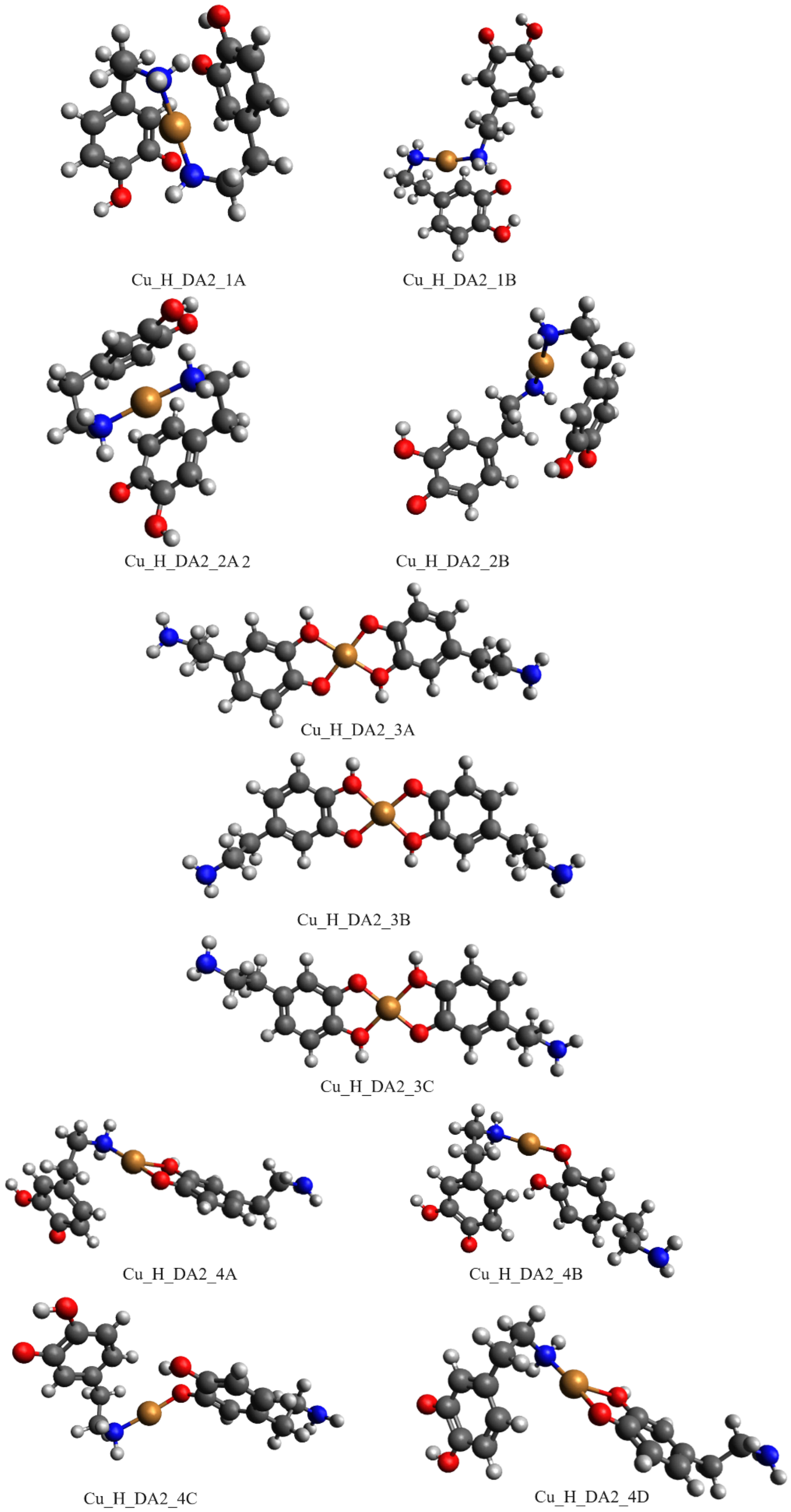

Figure S1. Optimized structures of obtained complexes consisted of two protonated molecules of dopamine and copper(II) ion.

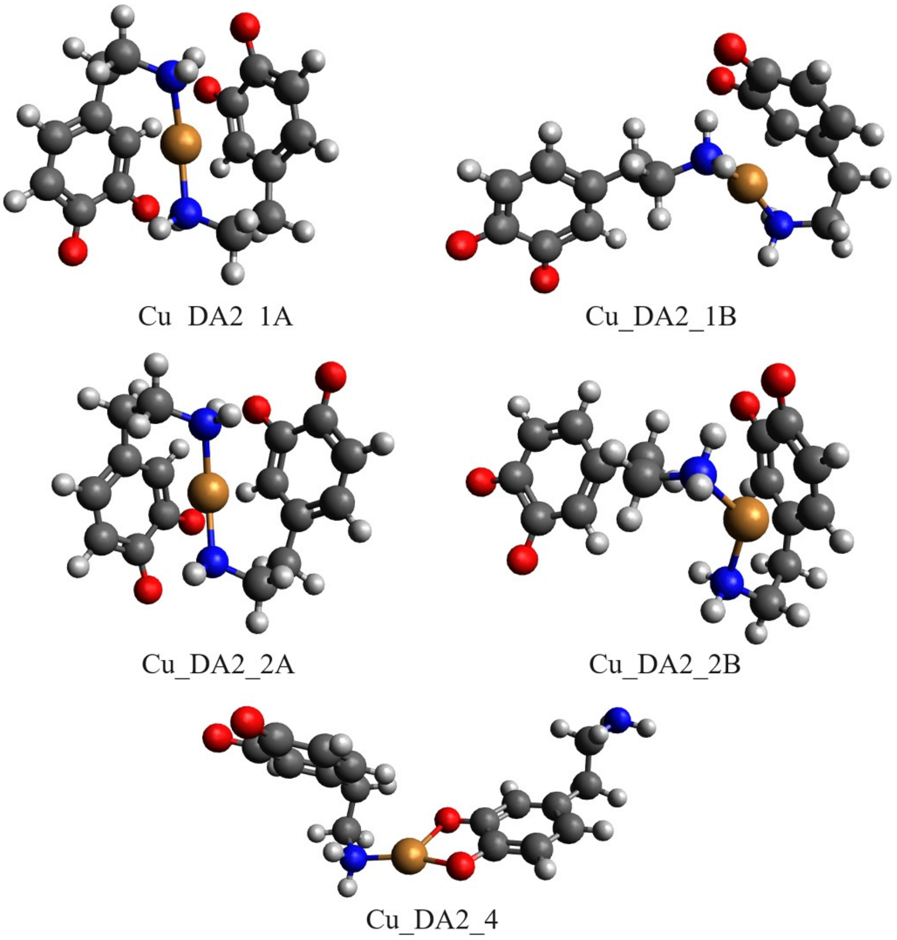

Figure S2. Optimized structures of obtained complexes consisted of two molecules of dopamine and copper(II) ion.

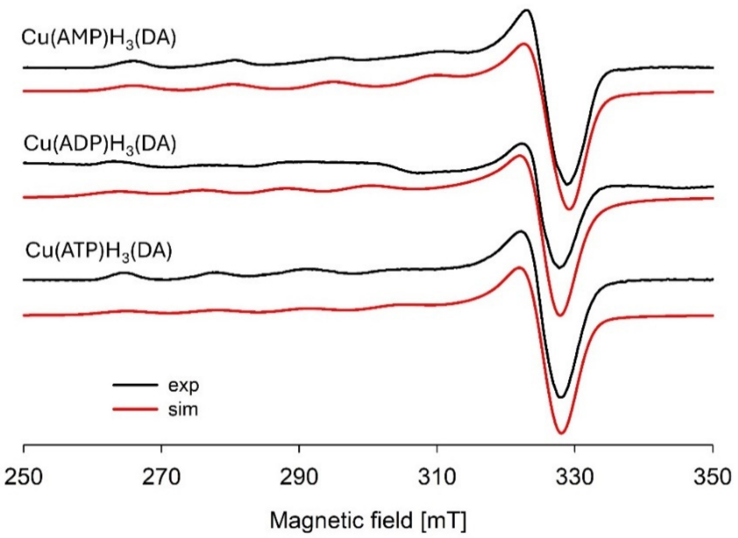

Figure S3. Experimental and simulated EPR spectra for the ML′H3L systems $C_{Cu^{2+}}=1\times10^{-3}$ M, $C_{L=L'}=2\times10^{-3}$ M.

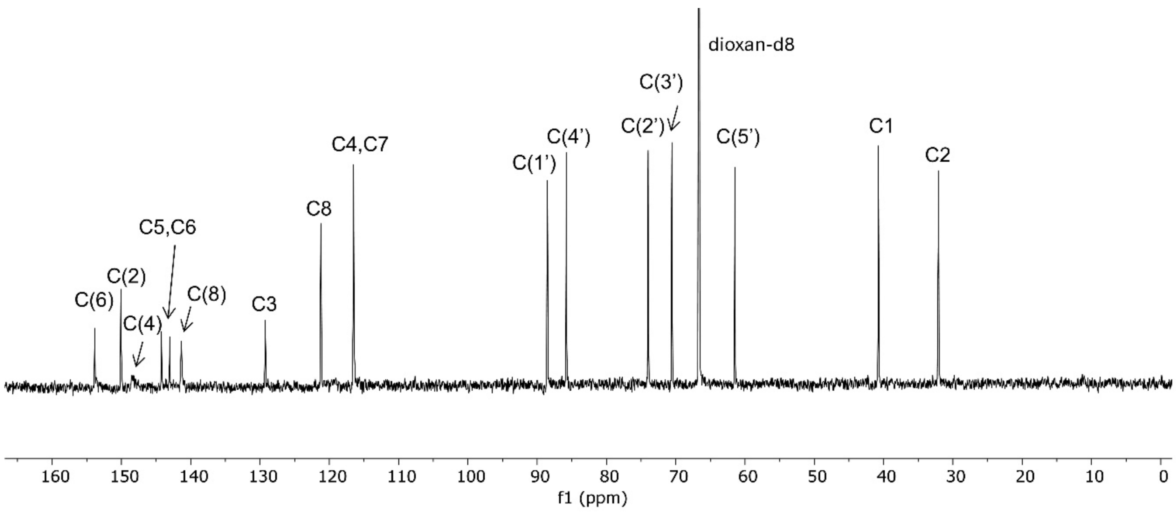

Figure S4. ^{13}C NMR spectra of Cu(II)/Ado/DA system at pH 4.0; $C_{Cu^{2+}}=1\times10^{-3}$ M, $C_{L=L'}=5\times10^{-2}$ M.

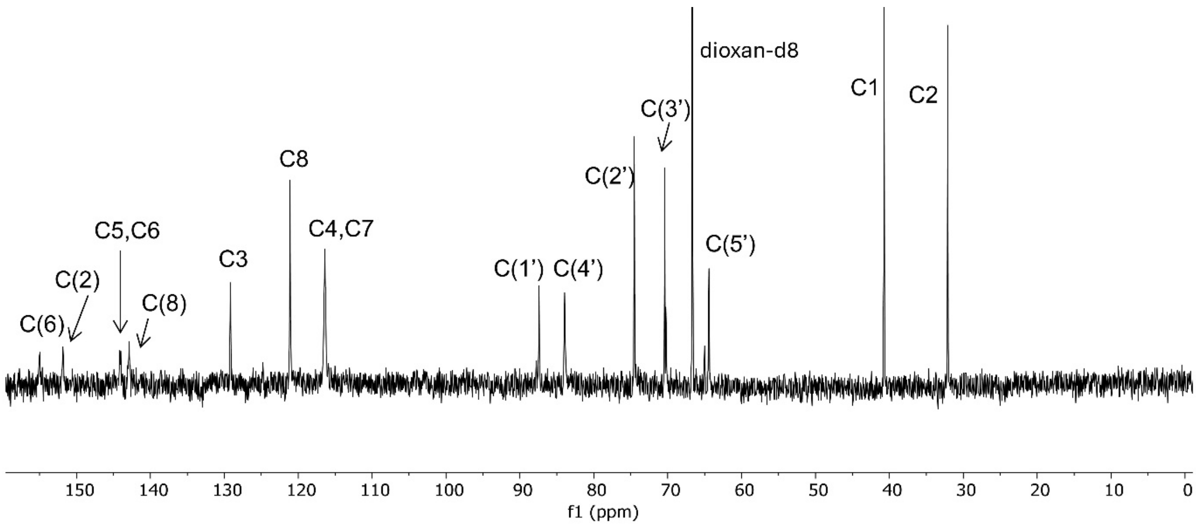

Figure S5. ^{13}C NMR spectra of Cu(II)/ADP/DA system at pH 5.1; $C_{Cu^{2+}}=1\times10^{-3}$ M, $C_{L=L'}=5\times10^{-2}$ M.

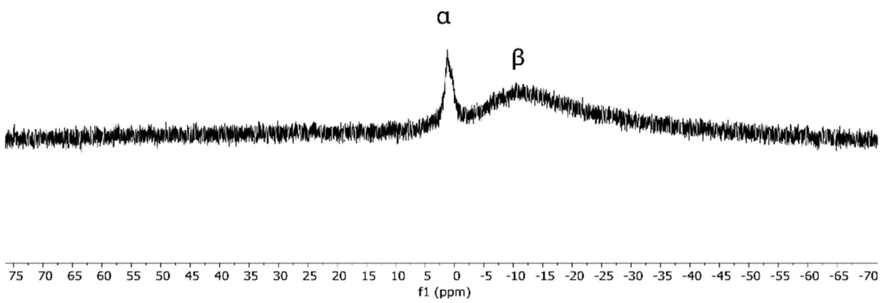

Figure S6. ^{31}P NMR spectra of Cu(II)/ADP/DA system at pH 5.1; $C_{Cu^{2+}}$=1×10^{-3} M, $C_{L=L'}$=5×10^{-2} M.

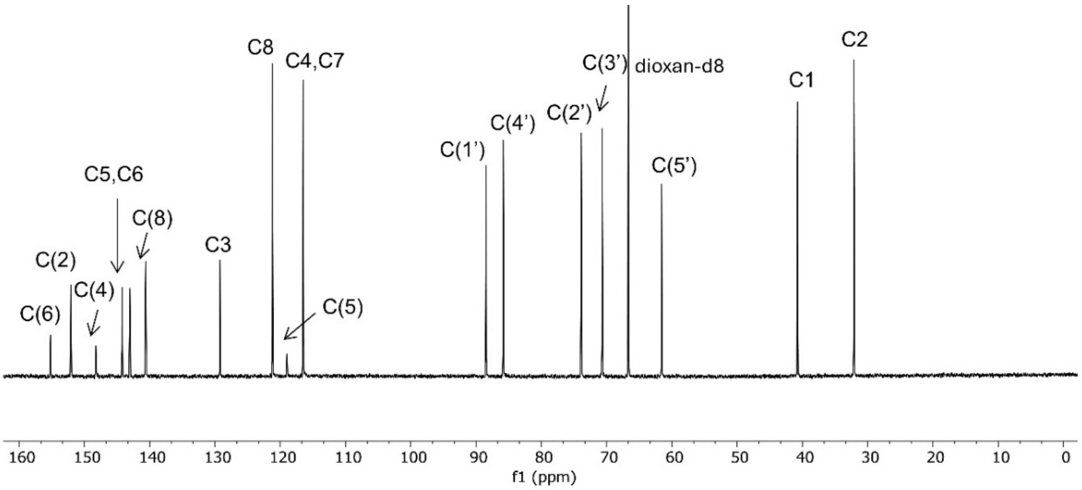

Figure S7. ^{13}C NMR spectra of Cu(II)/Ado/DA system at pH 5.1; $C_{Cu^{2+}}$=1×10^{-3} M, $C_{L=L'}$=5×10^{-2} M.

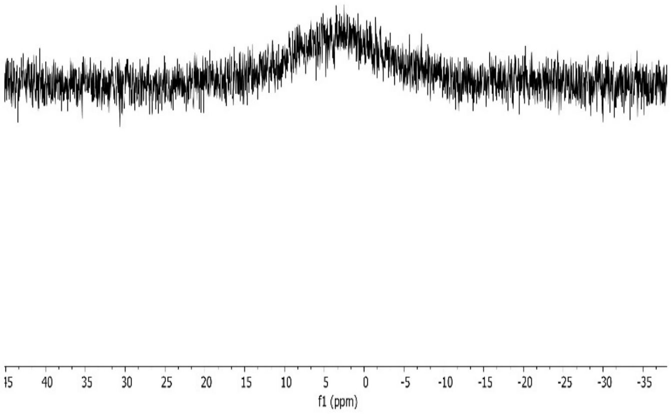

Figure S8. ^{31}P NMR spectra of Cu(II)/AMP/DA system at pH 6.8; $C_{Cu^{2+}}$=1×10^{-3} M, $C_{L=L'}$=5×10^{-2} M.

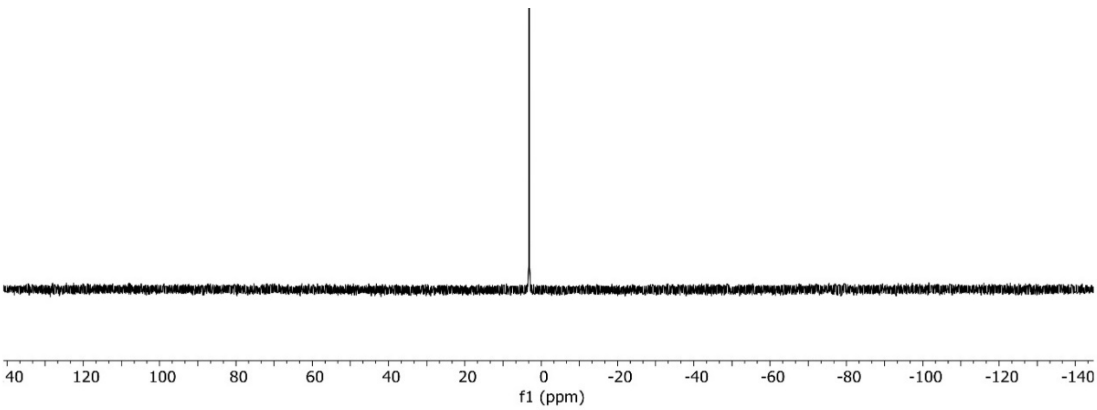

Figure S9. ^{31}P NMR spectra of AMP at pH 6.8; $C_{Cu^{2+}}$=1×10^{-3} M, $C_{L=L'}$=5×10^{-2} M.

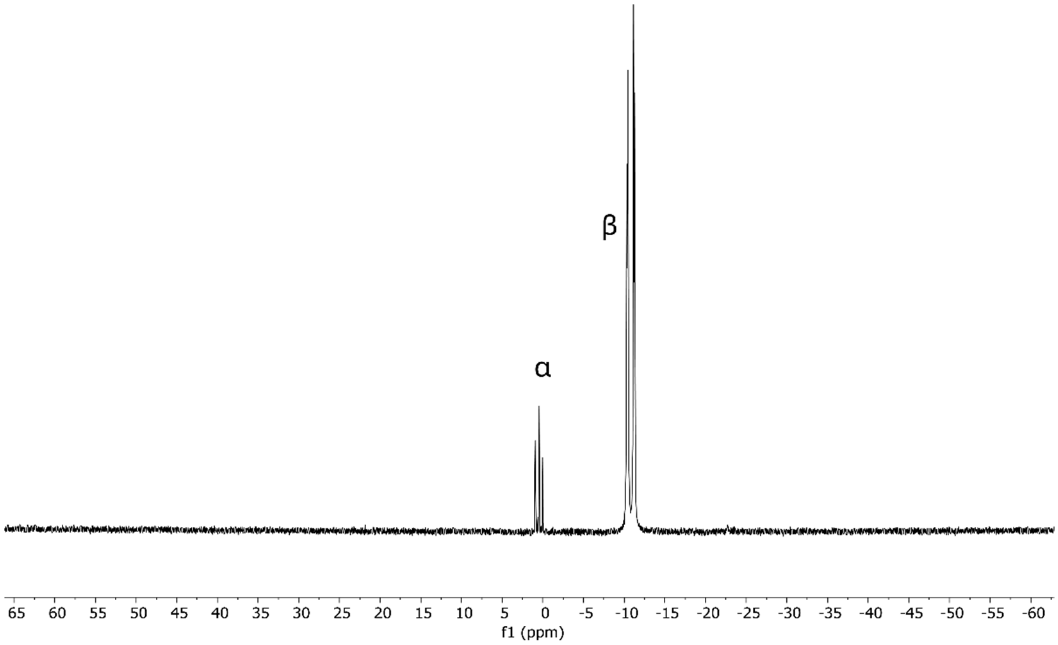

Figure S10. ^{31}P NMR spectra of ADP at pH 5.1; $C_{Cu^{2+}}$=1×10^{-3} M, $C_{L=L'}$=5×10^{-2} M.
